# An innovative intervention to improve respectful maternity care in three Districts in Ethiopia

**DOI:** 10.1186/s12884-021-03934-y

**Published:** 2021-08-06

**Authors:** Birkety Mengistu, Haregeweyni Alemu, Munir Kassa, Meseret Zelalem, Mehiret Abate, Befikadu Bitewulign, Kedest Mathewos, Kendra Njoku, Neil S. Prose, Hema Magge

**Affiliations:** 1 Institute for Healthcare Improvement, Ethiopia Project Office, Kirkos Sub-city, House No. 226, Addis Ababa, Ethiopia; 2 Federal Ministry of Health, Addis Ababa, Ethiopia; 3 Institute for Healthcare Improvement, Abuja, Nigeria; 4 Duke Global Health Institute, Durham, NC USA; 5 Division of Global Health Equity, Brigham and Women’s Hospital, Boston, MA USA

**Keywords:** Mistreatment, Disrespect, Abuse, Respectful maternity care, Quality improvement, Childbirth, Compassionate respectful care, Patient-centeredness, Experience of care, Ethiopia, Africa, Global health

## Abstract

**Background:**

Mistreatment of women during facility-based childbirth is a major violation of human rights and often deters women from attending skilled birth. In Ethiopia, mistreatment occurs in up to 49.4% of mothers giving birth in health facilities. This study describes the development, implementation and results of interventions to improve respectful maternity care.

As part of a national initiative to reduce maternal and perinatal mortality in Ethiopia, we developed respectful maternity care training module with three core components: testimonial videos developed from key themes identified by staff as experiences of mothers, skills-building sessions on communication and onsite coaching. Respectful maternity care training was conducted in February 2017 in three districts within three regions.

**Methods:**

Facility level solutions applied to enhance the experience of care were documented. Safe Childbirth Checklist data measuring privacy and birth companion offered during labor and childbirth were collected over 27 months from 17 health centers and three hospitals. Interrupted time series and regression analysis were conducted to assess significance of improvement using secondary routinely collected programmatic data.

**Results:**

Significant improvement in the percentage of births with two elements of respectful maternal care—privacy and birth companionship offered— was noted in one district (with short and long-term regression coefficient of 18 and 27% respectively), while in the other two districts, results were mixed. The short-term regression coefficient in one of the districts was 26% which was not sustained in the long-term while in the other district the long-term coefficient was 77%. Testimonial videos helped providers to see their care from their clients’ perspectives, while quality improvement training and coaching helped them reflect on potential root causes for this type of treatment and develop effective solutions. This includes organizing tour to the birthing ward and allowing cultural celebrations.

**Conclusion:**

This study demonstrated effective way of improving respectful maternity care. Use of a multipronged approach, where the respectful maternity care intervention was embedded in quality improvement approach helped in enhancing respectful maternity care in a comprehensive manner.

**Supplementary Information:**

The online version contains supplementary material available at 10.1186/s12884-021-03934-y.

## Background

Increasing access to skilled care during childbirth is a key strategy for reducing maternal and perinatal mortality and morbidity [1]. Mistreatment, however, is highly prevalent in health facilities globally and ranges from subtle negligence and abandonment to overt verbal or physical abuse [2–7]. The Bowser and Hill framework is commonly cited to describe seven categories of mistreatment during childbirth [4, 6, 8–12]. These include physical abuse, non-consented care (including denial of birth companionship), non-confidential care, non-dignified care (including verbal abuse), discrimination based on specific attributes, abandonment or denial of care and detention in facilities.

These forms of mistreatment are major human rights violations and discourage women from seeking care during birth [3, 4, 8, 9, 13–19]. In addition, mistreatment has been shown to negatively affect clinical outcomes [2, 6, 19, 20]. Mistreatment can occur at the level of interaction between women and health care providers (HCP) or may result from health system failures, including supply constraints or non-enabling facilities [3, 12, 21].

Ethiopia has a low facility-based birth rate of only 26% [22]. Systematic analyses of studies on mistreatment in Ethiopia have shown that among those who utilize facility-based care, almost half (49.4%) are experiencing neglectful or abusive care, with 13.6% reporting physical abuse and 16.4% abandonment [23]. Lack of training on interpersonal communication, poor working environments and high workloads are among the drivers of mistreatment [6, 13, 24]. In the long-run such mistreatment tends to be accepted as the ‘norm’ both by women and HCPs and may not be raised as concern [4, 9, 12, 25–27].

Although birth companions provide emotional, psychological and social support, birth companions were not widely allowed in Ethiopia. Recent studies have shown that the presence of a birth companion in the labor ward is associated with improved outcomes for both mother and baby, including increased spontaneous vaginal births, shortened labor time and higher Apgar scores [28]. In fact, women who felt that they received dignified and supportive care reported fewer newborn complications [19].

To address mistreatment in health care, the Federal Ministry of Health of Ethiopia (FMoH) focused on motivating health professionals to become Compassionate, Respectful and Caring (CRC) as one of the four priority agendas of the Health Sector Transformation Plan [29]. Although there is increasing documentation of effective interventions to reduce mistreatment globally [14, 30], there are no studies that have shown effective interventions to reduce mistreatment in maternity care in Ethiopia.

The Institute for Healthcare Improvement (IHI)^1^ in partnership with the FMoH, integrated an approach to institutionalize respectful maternity care (RMC) into a large-scale maternal newborn health (MNH) focused quality improvement program. The RMC intervention aimed to empower HCPs through a life-testimonial video-based training that also contained participatory discussion and reflection, didactic sessions on communication skills and onsite coaching to devise local solutions that can enhance RMC. This study describes its development and implementation and measures its effectiveness using programmatic data.

**Fig. 1:**
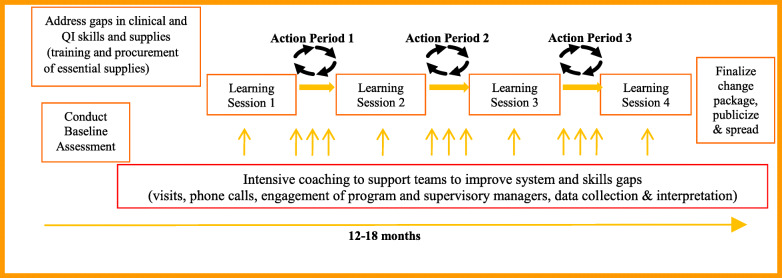
District-wide quality improvement approach of IHI

The MNH Quality Improvement Collaborative aims to reduce maternal and neonatal deaths by 30% through the introduction of a district-wide quality improvement (QI) program. Details of its design are published elsewhere [31]. In brief, the approach brings teams together from all facilities in the district for district-level improvement collaborative work for a period of 12–15 months. Target indicators representing key evidence-processes of MNH were selected by national and regional MNH leadership based on country-wide priorities, including two RMC-focused measures related to birth companionship and privacy. The program commenced in April 2016 with a facility leadership training in QI to build leadership buy-in, followed by baseline assessment of key MNH-care inputs, processes and outcomes using data from the previous 12 months (July 2015 to June 2016) to determine areas for improvement. During the first learning session, in November 2016, QI teams from all facilities in the district were convened, trained in QI methods and presented with the baseline findings. QI teams were then supported to design QI projects to fill gaps identified in the baseline assessment. These teams pursued collective aims of improving MNH by generating and testing new ideas, using multiple Plan-Do-Study-Act (PDSA) cycles in their local facilities during the action period between two learning sessions [32]. Intensive QI coaching and MNH clinical mentorship occurred during each action period (Fig. 1). When training gaps were found during baseline assessment, HCPs received basic emergency obstetric and newborn care (BEmONC) training which included RMC orientation.

IHI provided the QI training, the baseline assessment tool and supported facilities in conducting baseline assessment. Coaching visits to the facility teams were done by IHI senior project officers, while BEmONC training was provided by Ethiopian Midwives Association in collaboration with IHI. In close consultation with the regional health bureaus, three districts were targeted as prototype districts in Tigray, Oromia and Southern Nations, Nationalities, and People’s (SNNP) Regions (Table 1).

**Table 1:** Health facilities by residing population, birth volume and providers who participated in the training session, in the three districts

Region	Health Center				Hospital				Total Births in 27 months	Catchment Population
	Number	Participants	Birth Volume		Number	Participants	Birth Volume			
			Avg./month	Std. dev.			Avg./month	Std. dev.		
Tigray	5	23	25.6	13.9	1	11	72.5	10.3	5395	107,081
SNNP	5	27	48.2	17.5	1	7	89.4	8.4	8815	122,316
Oromia	7	29	27.7	15.0	1	10	126.6	15.6	8919	213,032
Total	**17**	**79**			**3**	**28**			**23,129**	**442,429**

### RMC intervention

#### Design of the RMC videos

We conducted a focus group discussion (FGD) with IHI senior project officers who had first-hand experiences as HCPs in rural settings. Focus group participants drew upon their experiences with supporting and listening to pregnant women during community engagement activities. They also had witnessed as clinical mentors and HCPs disrespect and abuse that women faced first-hand. In the FGD, we explored the current state of RMC-related issues in the program-supported districts. These findings were consolidated and key themes identified. Three testimonial scripts were written to capture these key themes, depicting a woman with an uncomplicated birth, another one with referral and emergency care, and an adolescent pregnant woman with preterm labor (Additional file 1). The scripts were three to four minutes long and translated into Amharic. Volunteer actresses were then trained to perform the scripts in video testimonials to protect confidentiality.

#### Delivery of the RMC training module

The videos were shown to participants during the second learning session. Participants in the three districts are described in Table 1. The session was attended by multidisciplinary health professionals, including facility leadership, MNH clinical providers, data managers and health extension workers. The three videos were followed by participatory reflection and discussion. Participants were asked to reflect on the videos using questions depicted in Additional file 2.

After discussion, a short presentation followed on the prevalence of mistreatment in Ethiopia and a skills-building session on empathic communication and relationship development with women (Additional file 2). Following the session, participants returned to their QI teams to develop new ideas or local solutions to enhance RMC in their facilities using multiple PDSA cycles. The importance of testing changes to enhance RMC was reinforced by facility coaches during coaching visits in the action period. These visits also helped to collect data and assure data quality. A minimum of three coaching visits happened per facility in each action period.

## Methods

### Data collection

Measures targeted for improvement through this intervention were privacy and birth companions offered during labor and childbirth. Monthly programmatic data indicating the percentage of births with privacy maintained and birth companion offered were collected from the 17 health centers and three primary hospitals in the three districts from November 2016 until January 2019 for a total of 27 months. Data were sampled from 30 births in the previous month that had been monitored using the FMoH-adopted Safe Childbirth Checklist (SCC). Systematic sampling was used for facilities that have higher numbers of births (Additional file 3- SCC). For facilities with lower births (30 or less), all SCCs filled-in during the past month were reviewed. IHI senior project officers collected the data and entered these into the program database as part of their routine work. Even though RMC training addressed all seven categories of mistreatment, only births with privacy and birth companion offered were documented in the database. Hence, we used these two measures to assess effectiveness of the training module. We only registered whether a companion during birth was offered and not whether a birth companion was actually present during birth.

New ideas tested in facilities were extracted from routine QI coach documentation and evaluated based on quantitative criteria for “success” based on run chart rules^2^ [32]. Those with higher degrees of success and with an RMC focus were extracted for this analysis.

### Data analysis

We conducted an interrupted time series and regression analysis using STATA version 13 to analyze the effectiveness of the intervention. In the regression analysis, we analyzed short-term effects of the intervention which measure the first 10 to 11 months following the training (February/March to December 2017 during which direct project support occurred), while long-term effects measure the impact of the intervention after direct support ended. We used the Bowser and Hill mistreatment categories to label a ‘change idea’ as having a component that aims to enhance RMC. We presented the new change ideas implemented in the facilities that successfully enhanced the experience of care for women as results.

## Results

### Quantitative results on privacy and birth companion offered during labor and birth

A total of 23,129 births took place during the 27 months from November 2016 to January 2019 in the 20 health facilities. On average, in each of 17 health centers 34 births were attended per month. In the three primary hospitals on average 96 births occurred per month (Table 1).

**Fig. 2:**
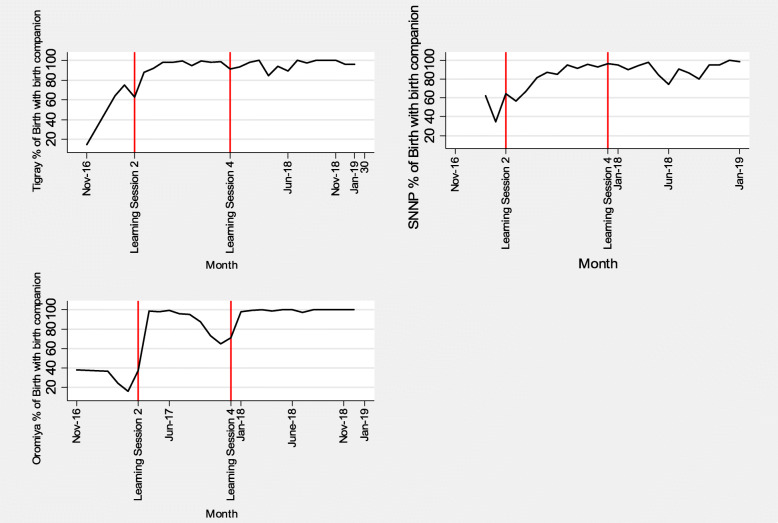
Percentage of births offered companionship in three regions in Ethiopia

**Fig. 3:**
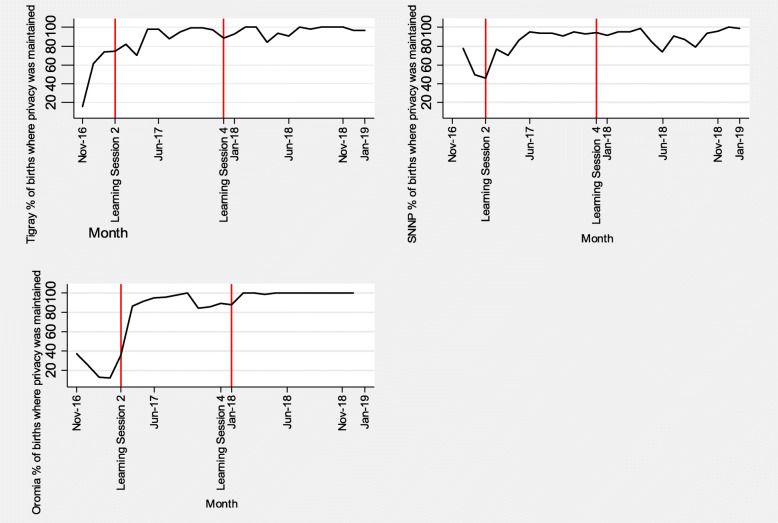
Percentage of births with privacy maintained in three regions in Ethiopia

Figure 2 shows an interrupted time series for the percentage of sampled births offered a birth companion by district. Figure 3 shows an interrupted time series analysis for the sampled percentage of births where privacy was maintained by district. The first vertical lines indicate when the second learning session in which RMC was introduced. This was conducted in the district of Oromia in February 2017 and in Tigray and SNNP in March 2017. Baseline data were collected from November 2016 (when the SCC was introduced) until February/March 2017. The second vertical line depicts the end of the direct project support (the fourth learning session) in December 2017, while data collection continued until January 2019.

As shown in Table 2, regression analysis in Tigray showed a significant increase in percentage of births with companion and privacy during the project period (increment trend of 18% from the baseline trend of reduction of 27%, *p* < 0.001) which was maintained in the long-term, a year after the end of the direct project support (increment trend of 27%, p < 0.001). In SNNP, there was a significant short-term effect on the percentage of births with companion and privacy (increment trend of 26% from the baseline increment trend of 1%, *p* = 0.02) while this was not reliably sustained in the long-term (with reduction trend of 1%, *p* = 0.94). In Oromia, the short-term effect was not as remarkable (with reduction trend of 46% from the baseline reduction trend of 78%, p = 0.02) while the long-term effect was significant with an increased percentage of births with companion and privacy (increment trend of 77%, *p* = 0.002).

**Table 2:** Regression analysis of births with companion offered and privacy maintained by region

**Tigray** births with companion and privacy	**Coef.**	**Std. Err.**	**T**	**P > t**	**[95% Conf. Interval]**	
Time	−0.27	0.06	−4.8	P < 0.001	− 0.38	−0.15
Short term intervention effect	0.18	0.05	4.06	0.001	0.09	0.28
Long term intervention effect	0.27	0.06	4.8	P < 0.001	0.15	0.38
Constant	1.85	0.22	8.39	P < 0.001	1.39	2.31
**SNNP** births with companion and privacy	**Coef.**	**Std. Err.**	**T**	**P > t**	**[95% Conf. Interval]**	
Time	0.01	0.07	0.18	0.86	−0.13	0.16
Short term intervention effect	0.26	0.11	2.46	0.02	0.04	0.48
Long term intervention effect	−0.01	0.07	−0.08	0.94	−0.15	0.14
Constant	0.48	0.22	2.22	0.04	0.03	0.94
**Oromia** births with companion and privacy	**Coef.**	**Std. Err.**	**T**	**P > t**	**[95% Conf. Interval]**	
Time	−0.78	0.23	−3.39	0.003	−1.25	−0.30
Short term intervention effect	−0.46	0.18	−2.55	0.019	−0.83	−0.09
Long term intervention effect	0.77	0.22	3.48	0.002	0.31	1.24
Constant	3.8	0.93	4.08	0.001	1.87	5.75

### Interventions deployed to enhance RMC

In addition to the outcome data, we assessed the new ideas tested in the facilities as part of improving the overall MNH quality of care. Out of a total of 73 new ideas tested by the QI teams, 27 were related to RMC, and among these, 23 met the criteria for inclusion in the change package. A list of successfully tested new ideas related to improving the experience of care of women is shown in Table 3. In all three districts, pregnant women conferences, where pregnant women come together monthly for group counselling, were modified to include discussions on new efforts to maintain women’s privacy during labor and birth and encourage women to bring a birth companion. A tour of the labor ward and a coffee ceremony were also incorporated into pregnant women conferences. To ensure privacy of women during birth, health facilities developed new ideas such as using screens, including those that are made from locally available materials.

**Table 3:** RMC related new ideas tested in the health facilities

**New idea** **(What?)**	**New Idea** **(How?)**
**Women’s engagement**: Enhancing the Pregnant Women Conference (PWC)	Enhance PWC by having **open discussion** with pregnant women about the continuum of care **and challenges they face**. **Pregnant women are encouraged to ask questions** and patients’ stories (near misses and complications) are heard. Such stories were obtained from the Maternal Death Surveillance and Response team. Women are requested to bring their antenatal care (ANC) appointment card. ANC lab tests were provided as appropriate by taking lab reagents and equipment to the PWC site.
	Numbers of pregnant women who attended PWC and **their feedback is documented**. Facility head analyzes the data and **feedback from women is shared with QI team weekly for decision making and as input in QI project design**.
	Content of messages during PWC were modified to include **birth position preparedness**, **birth attendant preference**, availability of lab investigation at no costs, **opportunity for birth companion during labor and birth**, **efforts to maintain women’s privacy**.
	During monthly PWC, **coffee ceremony** is added as courtesy to women.
**Reduce waiting time**: Give priority to pregnant women in card room and lab.	Health center head will inform/orientate **card room staff to give priority to ANC attendees**, and during health education of ANC attendees, **card room staff will withdraw all ANC cards first, reducing waiting time**. Setting a system to obtain pregnant women’s medical records in advance based on their appointment week (Some used 12 months labelled box to put mothers’ cards based on the appointment month while others used an Excel-based tracking system).
	Staff orientation, especially to midwives, to send full lab investigations for all pregnant women with ‘ANC’ written on the top of lab request form. **Lab technicians give priority** and take samples first from ANC attendees once the form is received.
**Reduce waiting time**: Avail ANC drugs in ANC room	Discussion was made among QI team members, reaching consensus to **make ANC drugs available in ANC room** to **eliminate pharmacy service point.** Prescription is collected at the end of the day and given to pharmacy department for drug management.
**Confidential care** by dedicating separate ANC rooms	**Dedicating separate rooms for ANC**, family planning and post-natal care in facilities where it used to be provided in a single room.
^a^**Birth Companion**: inform pregnant women about option of having a birth companion present during labor/birth	Midwives inform pregnant women of this service and encourage her to identify and inform a birth companion of her choice ahead of labor onset.
^a^**Presence of birth companion during labor and birth**	**The midwives allow laboring mothers to have a birth companion of their choice**. Mothers were informed of this change idea during monthly PWCs and ANC visits. The identified birth companion is informed on her/his role ahead of time.
**Making the facility clean and attractive**	Cleaning the health facility, wards and birth couch. Creating attractive environment by making the compound green with signage for different service points.
**Tour Labor and Birthing ward**	During fourth ANC visit, **tour of labor and birthing ward** is organized to help women get familiar with setting before labor. During highest turnout of ANC attendees (usually on market day), head of facility and midwife in charge host a tour to labor and birthing ward for those on the fourth visit. This usually lasts for 10–20 min where questions and comments are addressed. Names and numbers of attendees are registered in a designated template.
**Maternity waiting home (MWH)**^a^**- Creating home like environment:** cultural coffee ceremony, allowing prayers	During their stay pre-birth, they are looked after daily, vital signs checked and provision of structured lesson plans for health education. The health center provides **supplies for coffee ceremony**. **Prayers and cultural celebrations allowed**.
	Organize community contribution to **equip and supply MWH** on a regular basis.
	Growing cereals within health facility compound, used to **support running costs for MWH**.
^a^**Use screen during labor & birth process**	**Make bed screen available** and use it during examination in labor ward.
	**Use clean screen** in the labor room to provide privacy for women receiving care in birthing ward.
^a^**Use of reminder**	Posting the SCC on the wall of the labor ward which prompts to check for danger signs, to wash hands and use gloves during exams, **to encourage birth companion be present and maintain privacy during labor and birth.**
	Midwife in charge of birth room will **make SCC available**. When a laboring woman is admitted, **the duty midwife files SCC** in the women’s folder and uses SCC as a reminder of care to be given. As she gives care per SCC reminder, she ticks SCC to confirm care provision.
^a^**Use of Spot Checks** to Reduce Variation	**Daily retrospective monitoring of SCC use for every birth.** Duty midwife reviews daily birth cards. She checks if SCC was used and discuss with the team areas for improvement.
	**Onsite coaching of duty midwife by Medical director and MNCH head.** Medical director coaches nurse/midwife on the job once per week randomly to ensure SCCs are filled according to standards and existing scenario. They provide real-time feedback to nurse/midwives.
**Postnatal care home-like environment**: arrange coffee ceremony and provide porridge to women	It is customary to celebrate with coffee and eat porridge following birth. Hence, utensils and supplies for coffee ceremony and making porridge are provided to birth companion and other family members to celebrate together.
**Transporting mothers and newborns back home by ambulance**	Pregnant women in labor **and newly delivered mothers with their newborns are transported back to their home by ambulance** so that they are encouraged to give birth in health facilities for future births.

*PWC* pregnant women conference, *SCC* safe childbirth checklist

Maternity waiting home is a room within the health facility dedicated for pregnant mothers who live far from the facility to come and stay when their due date approaches until labor ensues

^a^Change ideas related to provision of birth companion of choice and privacy during labor and birth

During reflection and discussion following the videos, participants noted that the videos reflected the reality of women’s experience of care during birth and helped them see their care from their patients’ perspectives. Examples of disrespect mentioned in the videos that resonated with HCPs, included lack of cleanliness in the facilities and failure of HCPs to introduce themselves to women. Discussions with participants further revealed that testimonial videos appealed to their feelings and allowed them an opportunity to be ‘in their patients’ shoes’ and empathize with women.

## Discussion

Our study revealed that RMC-focused interventions embedded into a district-wide QI approach led to significant improvements of the two measures of RMC in Tigray and SNNP. Significant improvements were noted after 13 months of the end of the project in Tigray and Oromia. The combination of RMC training sessions, embedded into the QI initiative, helped HCPs both understand and address some of the systemic issues that contributed to mistreatment.

Failure to allow a family companion during institutional childbirth is one of the deterrents to utilization of maternity care in Ethiopia and other low- and middle-income countries [4]. HCPs who had received RMC training specifically recognized the importance of encouraging family support and companionship. This led to improvement in the practice of offering birth companionship in the intervention sites.

Less notable results were seen in both privacy and birth companion option data in Oromia from October to December 2017. This may have been due to civil unrest that took place during this time, affecting short-term effects. In SNNP, long-term effects may have been affected by supply shortages of SCC; in cases where data were not recorded, it was assumed that services were not offered.

Previous studies of RMC-related interventions have shown the importance of a multifaceted approach, including training on RMC and addressing barriers of RMC [14, 30, 33]. Studies in countries such as Kenya and Tanzania, using pre- and post-comparative evaluation designs, showed reductions in mistreatment, ranging from 7 to 66% [14, 33]. We were not able to show specific reductions in mistreatment. Our analysis, however, showed significant increase in births where privacy was maintained and birth companionship was offered following RMC training.

### Strengths and limitations

This study adds to the limited literature on successful strategies to improve RMC in sub-Saharan Africa in a sustainable manner. Our study also has important policy and programmatic implications in that QI methods can be applied to identify and address root causes of disrespect in MNH. In combination with focused RMC skills building, QI could address individual and system level barriers to RMC. As the intervention districts were distributed over the three agrarian regions of Ethiopia, findings may be generalizable to other agrarian contexts.

Our study has some important limitations. Ideally, we would have liked to interview women who gave birth recently as key informants to develop the video scripts. The videos, however, were made in the initial stage of the project when we had not yet built the necessary level of trust with the local communities and were in a relative position of power to patients. Therefore, we opted to interview project staff who had extensive clinical mentorship, patient support, and community engagement experience as key informants to develop personas to characterize a sample of positive and negative experiences with the health system. Scripts were developed based on reported issues and mistreatment witnessed or experienced in health facilities, which have later been confirmed by HCPs. Scripts of persona testimonials were developed based on these findings and volunteer actors recited the scripts to protect confidentiality of actual users. As the project matured, we were able to supplement the videos by inviting community representatives to learning sessions to share their reflections on the video content, their personal experiences and priorities. This process further helped us confirm that the experience portrayed in the videos had actually reflected the reality.

Since our analysis is based on available programmatic data, we could not use all seven Bowser and Hill’s categories of mistreatment. That would have required to interview women and observe their interactions with HCPs. That said, privacy and support by a companion were identified as priority problems by women in Ethiopian settings and hence selected by FMoH to be included in the Ethiopian SCC [13, 25, 34–36]. Despite this limitation, the RMC training module was specifically designed to address all seven mistreatment categories. In this study, we focused on ensuring privacy (non-confidential care) and allowing family companionship (non-consented care) due to availability of these data. Other domains of RMC may have been incorporated into change ideas across any of the target indicators. Further study exploring impact of interventions in all domains of RMC is warranted. Validated tools that help to measure women-centered maternity care are becoming increasingly available and can be used in the evaluation of intervention implementation [21].

Another important limitation is that the SCC showed only if a birth companion was offered but not their actual presence. Hence, we were not able to indicate how many of those offered have actually had a birth companion during labor and childbirth. It would be important to include a variable that measures the actual presence of a birth companion in the SCC and not just the proposition of such options. In addition, as data were collected from the SCC, shortages of the form in some health facilities led to lower measurement of coverage even if privacy and birth companionship were offered, contributing to underestimate impact. Finally, without a comparison district, it is possible that the results were related to other factors, including the national initiative on CRC [29] and attributing results to the RMC approach alone is difficult.

## Conclusion

This study suggests that integrating RMC training into a QI program is effective in improving RMC. Use of testimonial videos are especially helpful as they appeal to the heart and remind HCPs of their moral obligation to treat women with dignity. Embedding this intervention within an ongoing QI effort enabled HCPs to look deeper into the care process and to reform it in ways that are genuinely family- and women-centered. Complementing RMC training with onsite coaching helped in institutionalizing RMC in the targeted facilities. These interventions could be replicated in similar settings to ensure women get RMC they deserve. Further studies would be useful to evaluate impact of such interventions on person-centered maternity care comprehensively. It would especially be important to include views and experiences of childbearing women directly in making the videos. Finally, it would also be important to add a variable on the actual presence of a birth companion in the SCC to enable measurement of actual presence of birth companionship.

## Supplementary Information

**Additional file 1.** Scripts of the testimonial videos. Three different scripts on a mother with uncomplicated birth, another one with referral and emergency care and an adolescent pregnant woman who experienced preterm labor.

**Additional file 2.** Video facilitation guide. (PPTX 816 kb)

**Additional file 3.** Safe Childbirth Checklist adopted by the Ethiopian Ministry of Health.

## Data Availability

The dataset is readily available upon request with permission of IHI.

## Abbreviations

ANC: Antenatal Care
BEmONC: Basic Emergency Obstetric and Newborn Care
CRC: Compassionate and Respectful Care
FGD: Focus Group Discussion
FMoH: Federal Ministry of Health
HCP: Healthcare Providers
IHI: Institute for Healthcare Improvement
MNH: Maternal Newborn Health
PDSA: Plan-Do-Study-Act
QI: Quality Improvement
RMC: Respectful Maternity Care
SCC: Safe childbirth Checklist
SNNP: Southern Nations, Nationalities, and People’s

## Footnotes

1 Institute for Healthcare Improvement is a non-governmental organization based in Boston, MA that uses improvement science to advance and sustain better outcomes in health and health care across the world, including Ethiopia.

2 Run chart rules are rules that help QI team in deciding if a change idea has led to improvement by using these four rules. 1. Shift: six or more consecutive points above or below the median 2. Trend: five or more consecutive points all going up or all going down 3. Too few or too many runs and 4. Astronomical point. For further reading on run chart rules refer the improvement guide (ref. 32) Page 436.

## References

[1] Souza J, Tunçalp Ö, Vogel J, Bohren M, Widmer M, Oladapo OT, Say L, Gülmezoglu AM, Temmerman M (2014). Obstetric transition: the pathway towards ending preventable maternal deaths. BJOG.

[2] Downe S (2019). Focusing on what works for person-centred maternity care. Lancet Glob Health.

[3] Bohren M, Vogel J (2015). The mistreatment of women during childbirth in health facilities globally: a mixed-methods systematic review. PLoS Med.

[4] Bowser D, Hill K (2010). Exploring evidence for disrespect and abuse in facility-based childbirth. USAID Traction Project.

[5] Sen G, Reddy B, Iyer A, Heidari S (2018). Addressing disrespect and abuse during childbirth in facilities. Reproductive Health Matters.

[6] Rosen H, Lynam P, Carr C (2015). Direct observation of respectful maternity care in five countries: a cross-sectional study of health facilities in east and southern Africa. BMC Pregnancy Childbirth.

[7] Kruk ME, Gage AD, Arsenault C, Jordan K, Leslie HH, Roder-DeWan S, Adeyi O, Barker P, Daelmans B, Doubova SV, English M, García-Elorrio E, Guanais F, Gureje O, Hirschhorn LR, Jiang L, Kelley E, Lemango ET, Liljestrand J, Malata A, Marchant T, Matsoso MP, Meara JG, Mohanan M, Ndiaye Y, Norheim OF, Reddy KS, Rowe AK, Salomon JA, Thapa G, Twum-Danso NAY, Pate M (2018). High-quality health systems in the sustainable development goals era: time for a revolution. Lancet Glob Health.

[8] Wassihun B, Zeleke S. Compassionate and respectful maternity care during facility based child birth and women’s intent to use maternity service in Bahir Dar. Ethiopia BMC Pregnancy Childbirth. 2018;18. 10.1186/s12884-018-1909-8 PMC6038196 29986659

[9] Warren CE, Njue R, Ndwiga C, Abuya T (2017). Manifestations and drivers of mistreatment of women during childbirth in Kenya: implications for measurement and developing interventions. BMC Pregnancy Childbirth.

[10] Respectful Maternity Care: The Universal Rights of Childbearing Women. Washington DC: White Ribbon Alliance; 2011.

[11] Sando D, Abuya T, Asefa A, Banks KP, Freedman LP, Kujawski S, Markovitz A, Ndwiga C, Ramsey K, Ratcliffe H, Ugwu EO, Warren CE, Jolivet RR (2017). Methods used in prevalence studies of disrespect and abuse during facility based childbirth: lessons learned. Reprod Health.

[12] Banks KP, Karim AM, Ratcliffe HL, Betemariam W, Langer A (2018). Jeopardizing quality at the frontline of healthcare: prevalence and risk factors for disrespect and abuse during facility-based childbirth in Ethiopia. Health Policy Plan.

[13] Shiferaw S, Spigt M, Godefrooij M, Melkamu Y, Tekie M (2013). Why do women prefer home births in Ethiopia?. BMC Pregnancy Childbirth.

[14] Kujawski SA, Freedman LP, Ramsey K (2017). Community and health system intervention to reduce disrespect and abuse during childbirth in Tanga Region, Tanzania: A comparative before-and-after study. PLoS Med.

[15] Asefa A, Bekele D (2015). Status of respectful and non-abusive care during facility-based childbirth in a hospital and health centers in Addis Ababa, Ethiopia. Reprod Health.

[16] Jackson R, Hailemariam A (2016). The role of health extension Workers in Linking Pregnant Women with Health Facilities for delivery in rural and pastoralist areas of Ethiopia. Ethiop J Health Sci.

[17] Adinew Y, Assefa N. Experience of facility based childbirth in rural Ethiopia: an exploratory study of Women’s perspective. J Pregnancy. 2017. 10.1155/2017/7938371 PMC5735784 29359048

[18] Miller S, Lalonde A (2015). The global epidemic of abuse and disrespect during childbirth: history, evidence, interventions, and FIGO’s mother−baby friendly birthing facilities initiative. Int J Gynecol Obstet.

[19] Sudhinaraset M, Landrian A, Afulani PA, Diamond-Smith N, Golub G (2020). Association between person-centered maternity care and newborn complications in Kenya. Int J Gynecol Obstet.

[20] Sheferaw ED, Bazant E, Gibson H, Fenta HB, Ayalew F, Belay TB, Worku MM, Kebebu AE, Woldie SA, Kim YM, van den Akker T, Stekelenburg J (2017). Respectful maternity care in Ethiopian public health facilities. Reprod Health.

[21] Afulani PA, Diamond-Smith N, Phillips B, Singhal S, Sudhinaraset M (2018). Validation of the person-centered maternity care scale in India. Reprod Health.

[22] Ethiopia Demographic and Health Survey 2016. Central statistical agency, Addis Ababa Ethiopia; the DHS program ICF Rockville, Maryland, USA. 2017.

[23] Kassa ZY, Husen S (2019). Disrespectful and abusive behavior during childbirth and maternity care in Ethiopia: a systematic review and meta-analysis. BMC Res Notes.

[24] Burrowes S, Holcombe SJ, Jara D, Carter D, Smith K. Midwives’ and patients’ perspectives on disrespect and abuse during labor and delivery care in Ethiopia: a qualitative study. BMC Pregnancy Childbirth. 2017;17(1). 10.1186/s12884-017-1442-1 PMC5567643 28830383

[25] Molla M, Muleta M, Betemariam W, Fesseha N, Karim A (2017). Disrespect and abuse during pregnancy, labour and childbirth: a qualitative study from four primary healthcare centres of Amhara and southern nations nationalities and People’s regional states. Ethiopia Ethiop J Dev.

[26] Betron ML, McClair TL, Currie S, Banerjee J (2018). Expanding the agenda for addressing mistreatment in maternity care: a mapping review and gender analysis. Reprod Health.

[27] Sen G, Reddy B, Iyer A (2018). Beyond measurement: the drivers of disrespect and abuse in obstetric care. Reprod Health Matters.

[28] Bohren MA, Hofmeyr GJ, Sakala C, Fukuzawa RK, Cuthbert A (2017). Continuous support for women during childbirth. Cochrane Database Syst Rev.

[29] Ethiopia-health-system-transformation-plan.pdf [Internet]. [cited 2019 Apr 18]. Available from: https://www.globalfinancingfacility.org/sites/gff_new/files/Ethiopia-health-system-transformation-plan.pdf.

[30] Ratcliffe HL, Sando D, Lyatuu GW, Emil F, Mwanyika-Sando M, Chalamilla G, Langer A, McDonald KP (2016). Mitigating disrespect and abuse during childbirth in Tanzania: an exploratory study of the effects of two facility-based interventions in a large public hospital. Reprod Health.

[31] Magge H, Kiflie A, Nimako K, Brooks K, Sodzi-Tettey S, Mobisson-Etuk N, Mulissa Z, Bitewulign B, Abate M, Biadgo A, Alemu H, Seman Y, Kassa M, Barker P, Burrsa DG (2019). The Ethiopia healthcare quality initiative: design and initial lessons learned. Int J Qual Health Care.

[32] Gerard J, Langley G, Ronald D (2009). The improvement guide: a practical approach to enhancing organizational performance.

[33] Abuya T, Ndwiga C, Ritter J, Kanya L, Bellows B, Binkin N, Warren CE (2015). The effect of a multi-component intervention on disrespect and abuse during childbirth in Kenya. BMC Pregnancy Childbirth..

[34] Teferra AS, Alemu FM, Woldeyohannes SM (2012). Institutional delivery service utilization and associated factors among mothers who gave birth in the last 12 months in Sekela District, north west of Ethiopia: a community - based cross sectional study. BMC Pregnancy Childbirth..

[35] Odo DB, Shifti DM (2014). Institutional delivery service utilization and associated factors among child bearing age women in Goba Woreda. Ethiop J Gynecol Obstetrics.

[36] Gebremichael MW, Worku A, Medhanyie AA, Berhane Y (2018). Mothers’ experience of disrespect and abuse during maternity care in northern Ethiopia. Glob Health Action.

