# Supplementary material for: An innovative intervention to improve respectful maternity care in three Districts in Ethiopia
Source: BMC Pregnancy Childbirth. 2021 Aug 6;21:541. doi: 10.1186/s12884-021-03934-y (PMC8343890; doi:10.1186/s12884-021-03934-y)
Supplement: Supplementary file 1 — Additional file 1. Scripts of the testimonial videos. Three different scripts on a mother with uncomplicated birth, another one with referral and emergency care and an adolescent pregnant woman who experienced preterm labor. [file 12884_2021_3934_MOESM1_ESM.docx]

**Additional file 1: Scripts of the testimonials**

**A mother with normal delivery**

The women in my village do not share very good experiences with the health center near my home. My cousin went there last year to have her baby. She walked many hours in pain to get to the health center, but when she arrived, there was no staff there at all. In the end, she had her baby alone with the help of a cleaner. The baby almost died but the nurse came in shortly. When I became pregnant, my health extension worker encouraged me to try going to the health center for antenatal care and the delivery, so I decided to try.

My ANC visit was ok, the nurse was nice to me, and told me many times that I had to come for the delivery. So, when I first felt labor pains, I told my mother and husband, and they helped me get to the health center by cart. When we arrived, my mother and husband were told they had to wait outside. I was left alone on the bed for hours while I went through a very painful labor. Every half hour or so, a male midwife would come to examine me. He never introduced himself. He never asked me my name.

There were 5 women in one room and no curtains separating the beds. At least the other women were with me in this same experience. Sometimes I wanted to ask for help, but there was no one there to help. The midwife and nurse sometimes were chatting on their phones and were busy doing some other tasks.

Finally, I got to time for pushing. I was terrified of the pain and I knew nothing about what was going to happen. The midwife returned but did not look me in the eye. He did not ask my name nor did he ever explain to me why the labor was taking so long.

Several times when I cried, the midwife slapped me and told me to keep quiet. Thank God, finally my baby came. I lay eagerly waiting to see my baby, but she disappeared for a long time. I could hear the nurses chatting on the phone, so I assumed the baby was ok.

No one spoke to me or let me see or hold her. Finally, the nurse brought her to me and I looked in my baby’s eyes for the first time. The nurse helped me put her to my breast and told me to breastfeed her. I was a little worried she was cold, so I put blankets around her.

The nurse told me I should stay at the health center for 24 hours. I stayed for a few hours, but no nurse saw me, there was little porridge and it was cold, and there was no coffee ceremony. I did not see much reason to stay so I told her I wanted to go home, and she did not seem to care. I am not sure I want to go there again.

**Referral and Emergency Care**

I am getting older now, and I have three sons and two daughters. The last child died was born at home and died the next day, so I decided to go to the health center this time. My mother in law and the traditional birth attendant argued with me, but I insisted that I was going to the health center and my health extension worker agreed.

The health extension worker had urged me to go to the maternity waiting home before I reached term. However, I had heard so many negative comments about these homes. There are not enough beds, food or water. I also didn’t have anyone else to take care of my other children.

As soon as I felt labor pains, I went to the health center. An old midwife listened to my abdomen and started whispering to the nurse. They looked at each other and turned to me with a frightened look.

Then they told me I was going to need an operation to get the baby out, so I needed to go to the hospital. They did not tell me why I couldn’t give birth naturally or why I needed to have an operation. The home birth attendants are always so calm. This midwife seemed like he had no experience.

I knew that I would be required to cover the cost of the ambulance but I didn’t have that money, so I was worried. I had never been to the hospital, but I had heard that it is very expensive. I was embarrassed to ask about those details again, so I did not mention my financial worries to the midwife. When the midwife saw that I was second-guessing my decision to go to the hospital, he told me that the baby would die if I didn’t go so I agreed.

The ambulance arrived 2 hours later. It did not feel like anyone was moving quickly. I went into the ambulance and traveled on a bumpy road for another hour to the primary hospital. The driver had to drive through people’s farm lands because there were no roads. It was so painful and they said there was no room for my husband to accompany me in the ambulance. I didn’t know how he would get there but he said he would meet me there.

I arrived at the hospital. Some people talked to each other and I was moved to the maternity ward. I was in too much pain to understand what was happening around me. There was someone in a white coat, I think a doctor who looked at my file and told the nurses that I would need an operation. Before I knew it, I was moved into another room. They never asked my permission to do the operation. I really had no choice.

When I woke up, I did not know if my baby was dead or alive, and I didn’t know where my family was. Finally, the doctor came in and told me that my baby had died. He told me that it was my fault; I didn’t take good care of my pregnancy and I didn’t arrive on time to the hospital. The next day they sent me home. In addition to the sorrow, I am suffering with pain. I do not know how to tell my family what happened.

**Adolescent mother with a preterm labor**

When I fell pregnant, I was very scared. I did not know what to do. He had promised to marry me, but he disappeared. My dad was so angry with me, so he doesn’t want to see my face since then. My mom though she is angry at me, she doesn’t have the gut to fully reject me because I am her daughter. Even then, I did not know what to do so I spent a majority of my time at home.

When I started having labor pains, I was extremely frightened because it was not the right time. I was praying it was not coming, but then the pain became stronger and I called out to my mother. I still had 2 months to go. I did not want to die and I did not want my baby to die.

My mother brought the health extension worker to our house. She said that I should go to the hospital as soon as possible. I had never been to that town before. My mother was not sure about it. She said even if the baby died, it was too early and would go to heaven. This scared me even more.

Finally the health extension worker convinced her and called the ambulance. It took 3 hours before it came and I was so scared because I thought I would have to give birth at home. The trip to the hospital was another 2 hours by ambulance. When we got there, we did not know what to do or who to talk to. Finally, a man in a white coat talked to my mother but did not look at me. He did not introduce himself. When he talked to my mother, he was not very friendly.

Then the midwife Beza arrived. She was much better than the doctor. She checked on me whenever she could and made sure to ask me what I needed. She gave me water and porridge as well. When she allowed my mother to sit with me, that made me feel safe.

I was in a lot of pain the whole time, and nobody was able to help me with that. In addition, I had no privacy at all. There were no curtains and everyone in the vicinity could look at me. A lot of students were just standing around and watching. It seemed like no one was helping.

But there was running water, and the facility was clean and Beza always washed her hands before she touched me. She also told me there were doctors and nurses who knew how to take care of babies born early so I was somewhat at ease.

Soon it was time to push. When the baby came out, she was quiet. Beza cut the cord, took the baby to a table next to her and gave her some treatments. It was then that I heard the baby crying. She brought her to me immediately and put her on my skin. She told me to keep her skin next to mine to keep her warm and put a hat on her head.

I was so scared because my baby was so small, but Beza smiled and held my hand and told me she would be ok. Her lungs were strong but she needed more care in the hospital. She told me to rest for an hour and then go to the room where the baby would be waiting for me and getting good care from the doctors and nurses. Thank God, she was right.
