# Supplementary material for: An innovative intervention to improve respectful maternity care in three Districts in Ethiopia
Source: BMC Pregnancy Childbirth. 2021 Aug 6;21:541. doi: 10.1186/s12884-021-03934-y (PMC8343890; doi:10.1186/s12884-021-03934-y)
Supplement: Supplementary file 2 — Additional file 2. Video facilitation guide. (PPTX 816 kb) [file 12884_2021_3934_MOESM2_ESM.pptx]

## Slide 1
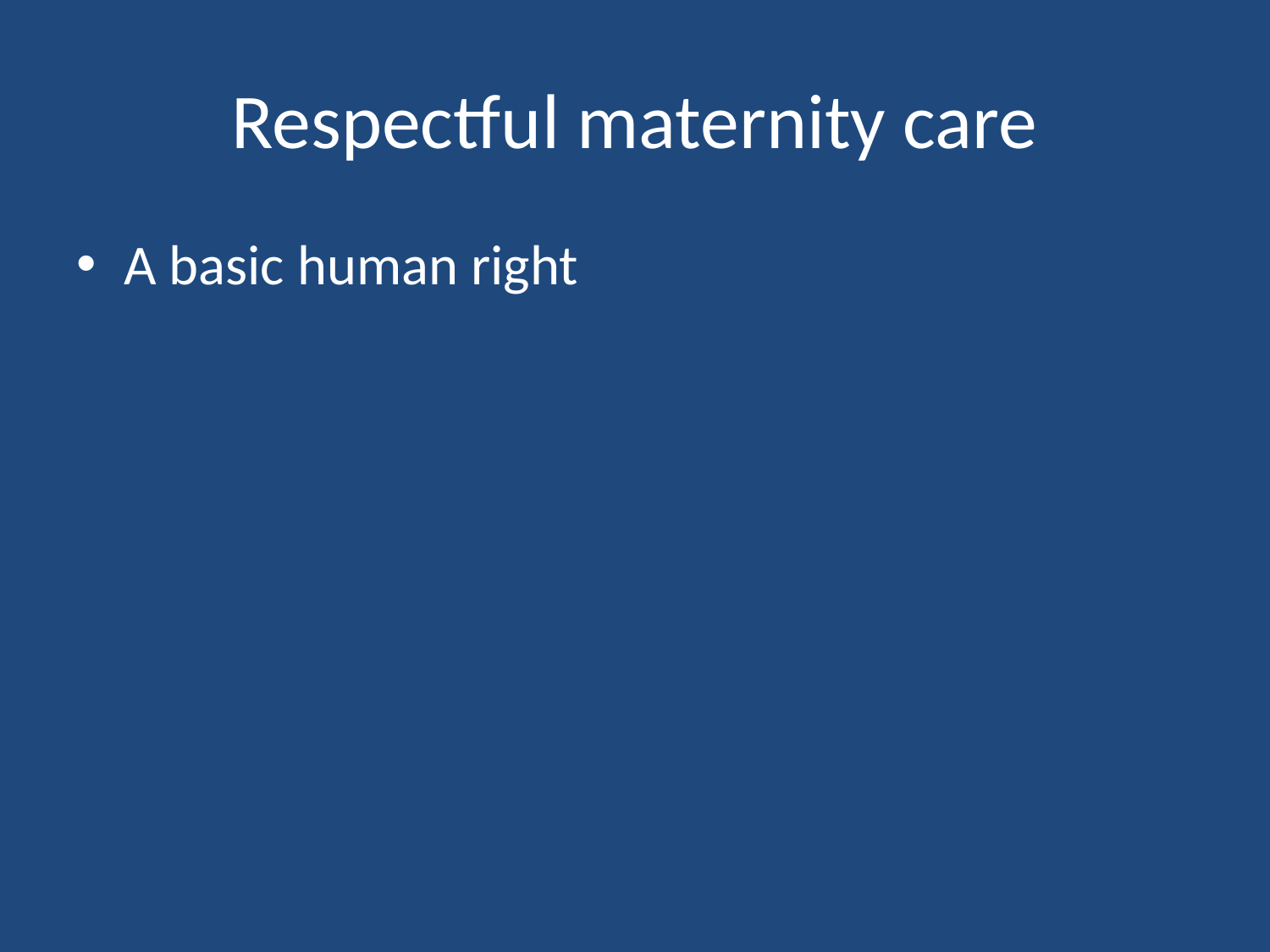

# Respectful maternity care
A basic human right

## Slide 2
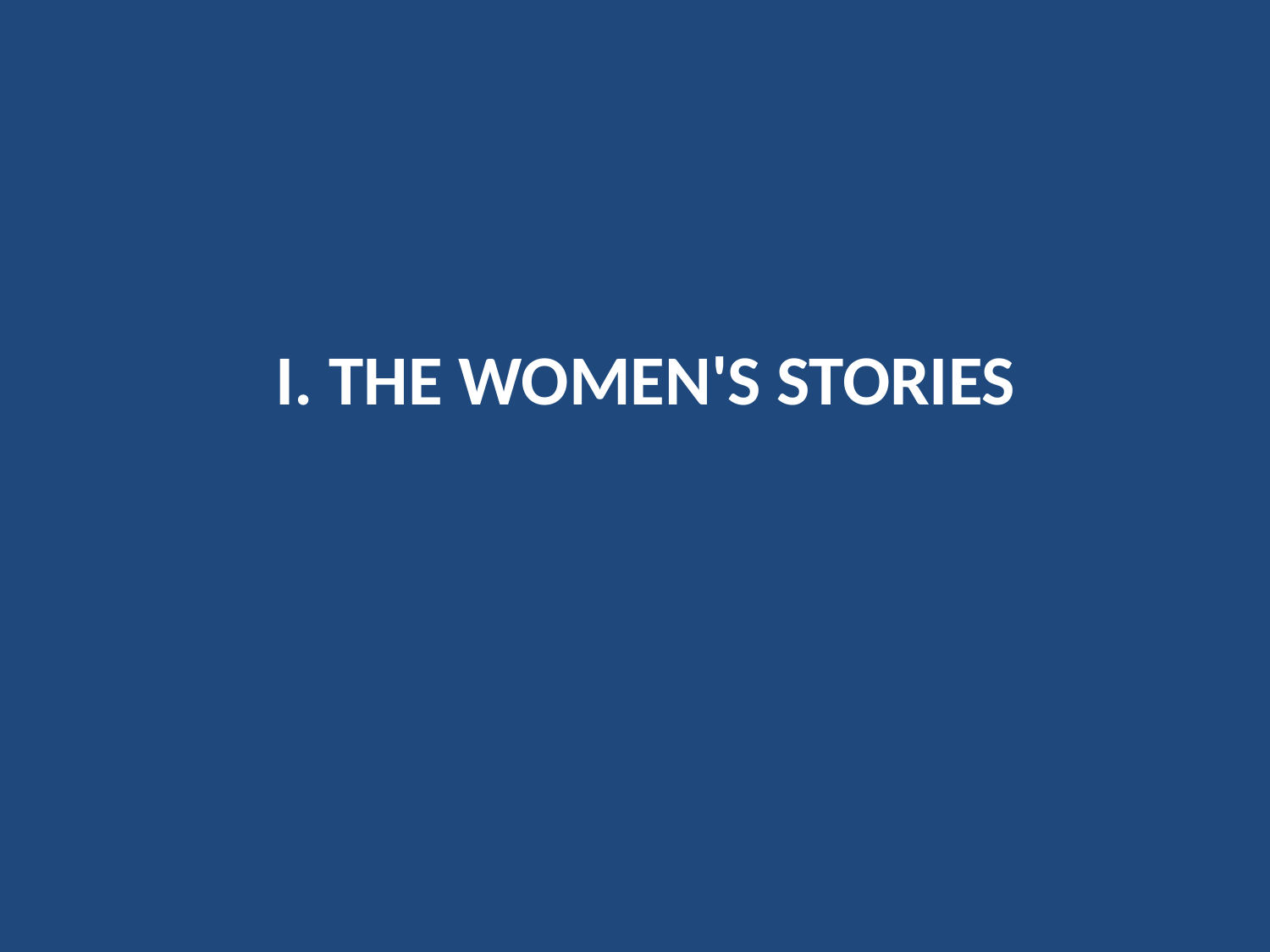

# I. The women's stories

## Slide 3
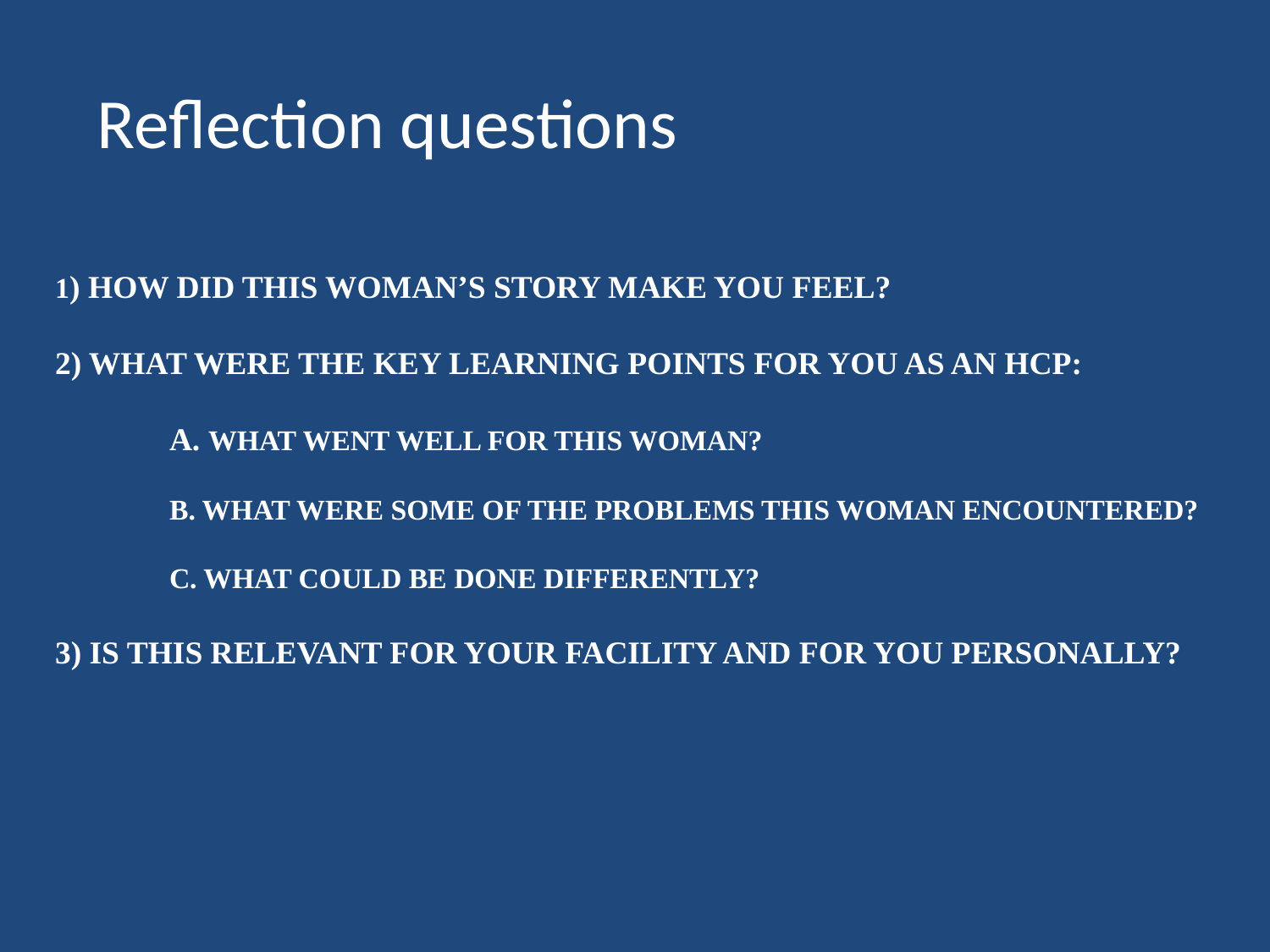

Reflection questions
# 1) How did this woman’s story make you feel? 2) What were the key learning points for you as an HCP: a. What went well for this woman? b. What were some of the problems this woman encountered? c. What could be done differently? 3) Is this relevant for your facility and for you personally?

## Slide 4
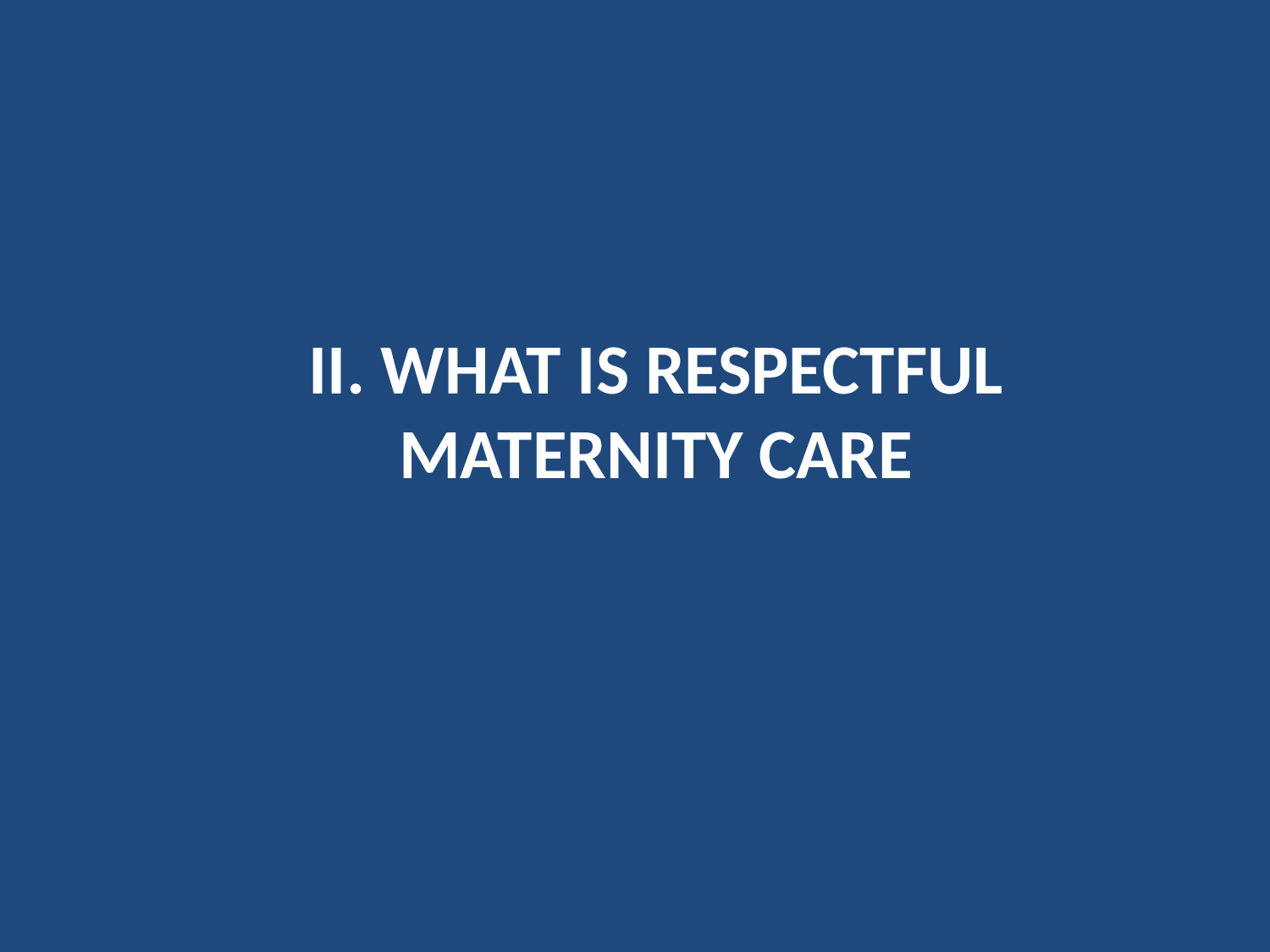

# II. What is respectful maternity care

## Slide 5
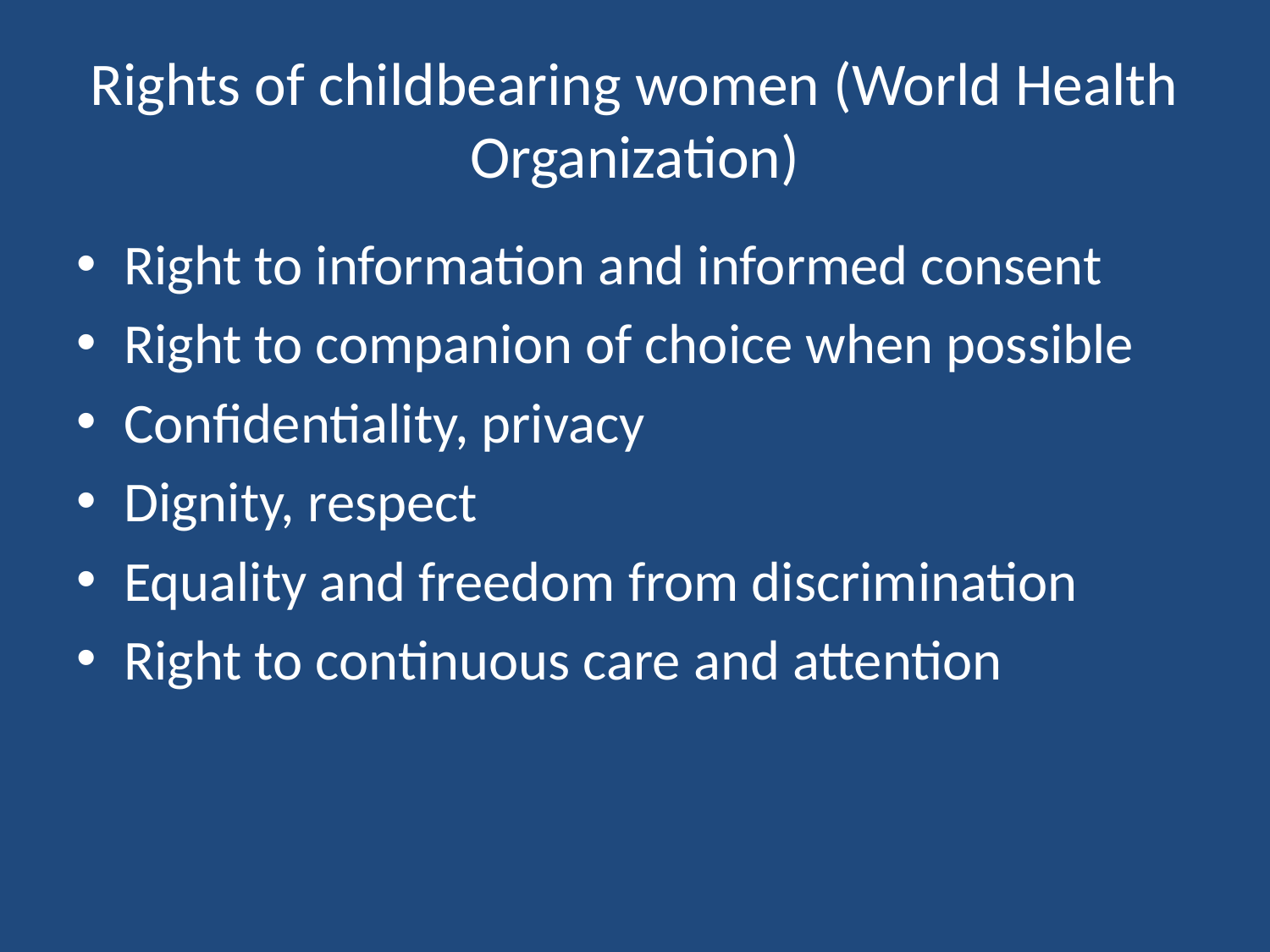

# Rights of childbearing women (World Health Organization)
Right to information and informed consent
Right to companion of choice when possible
Confidentiality, privacy
Dignity, respect
Equality and freedom from discrimination
Right to continuous care and attention

## Slide 6
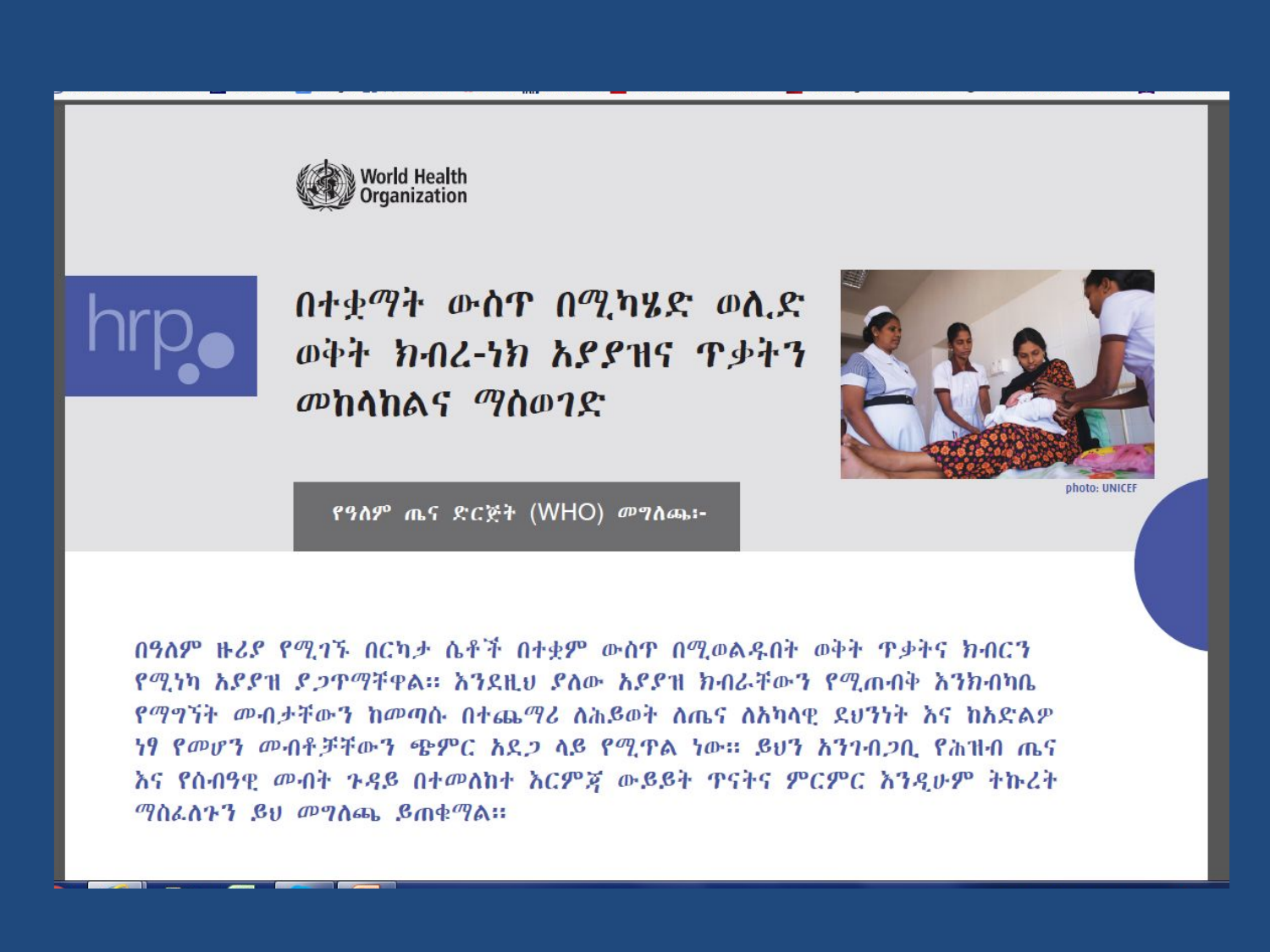

#

## Slide 7
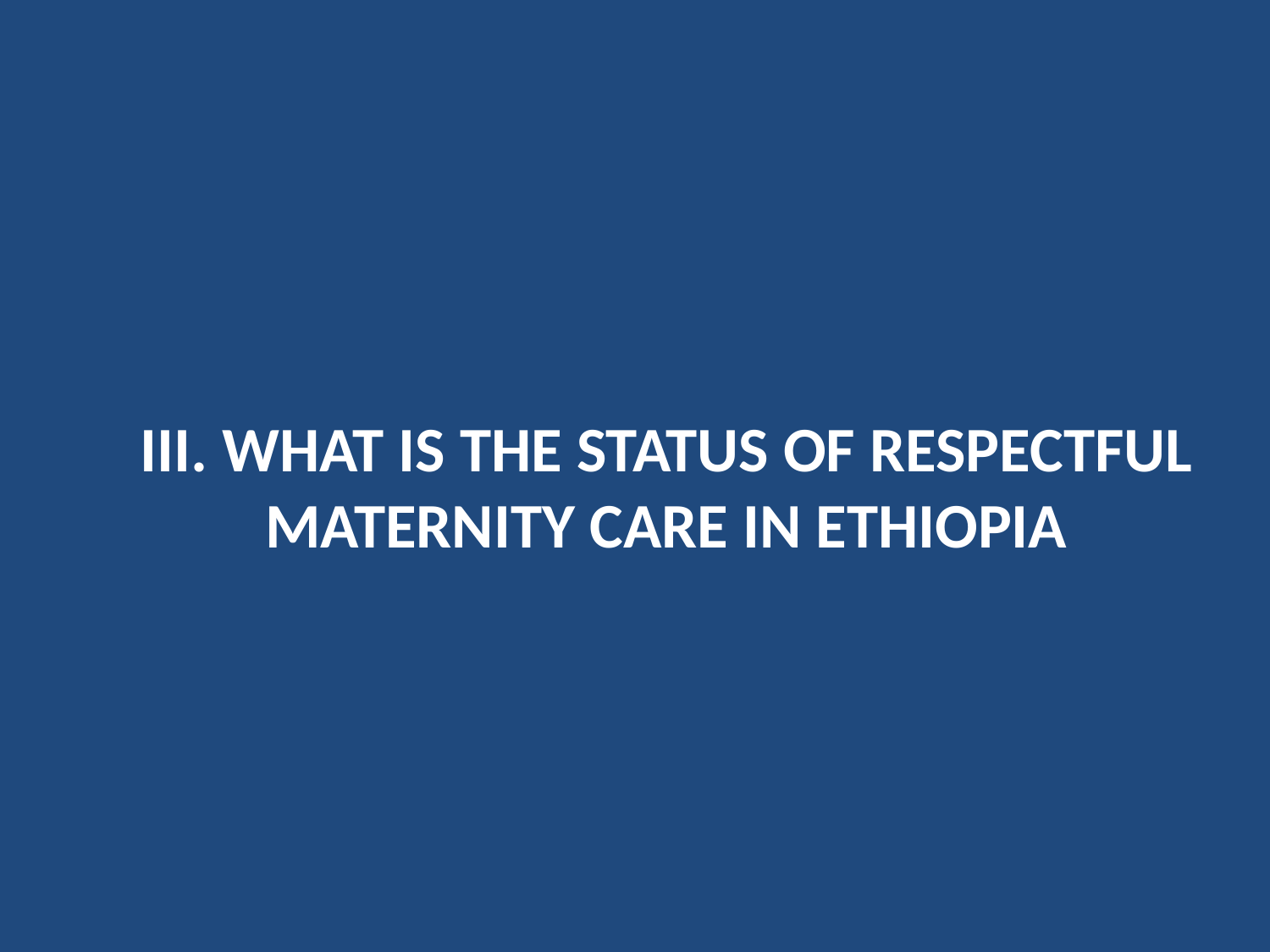

# III. What is the status of respectful maternity care in Ethiopia

## Slide 8
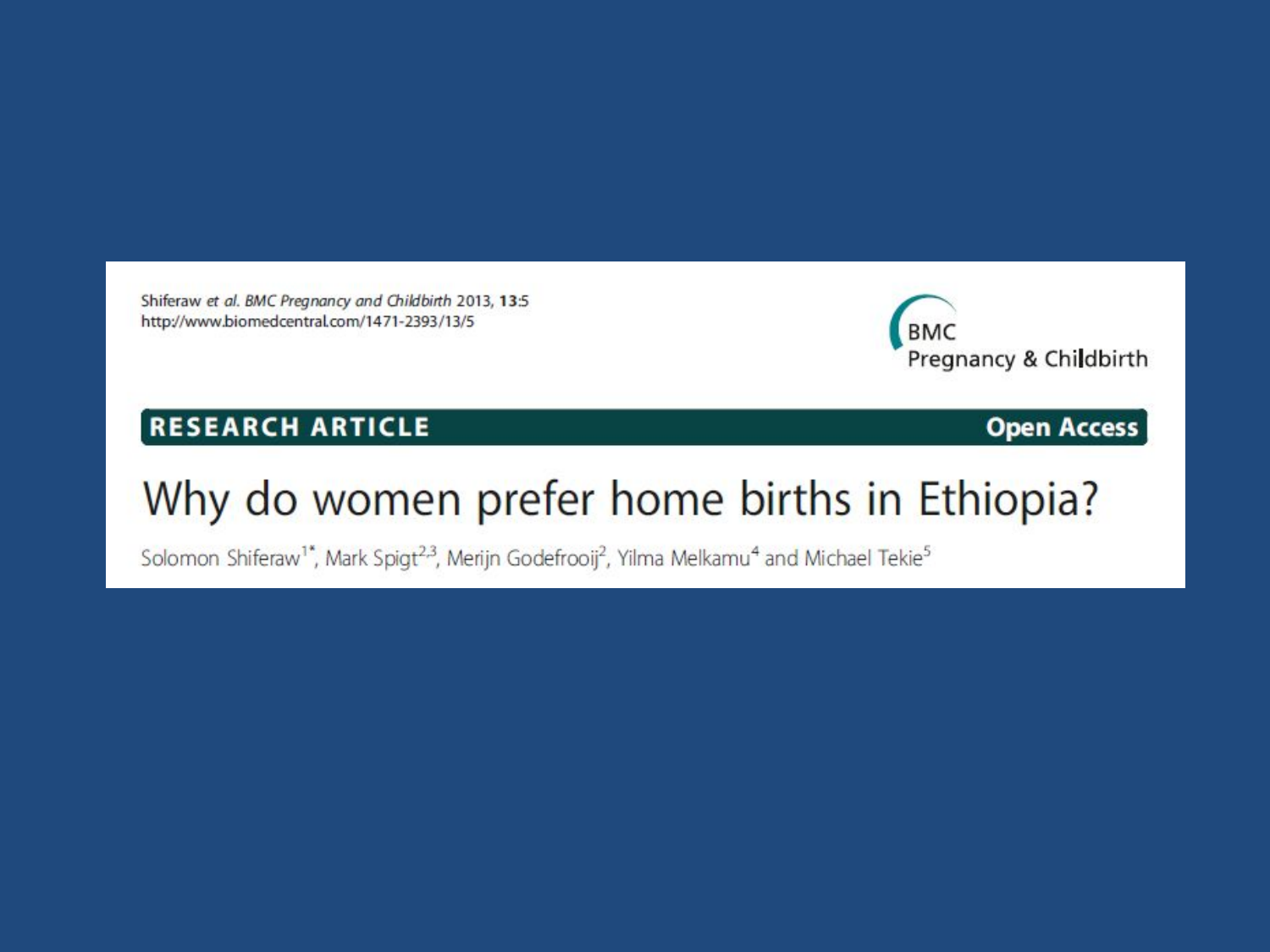

## Slide 9
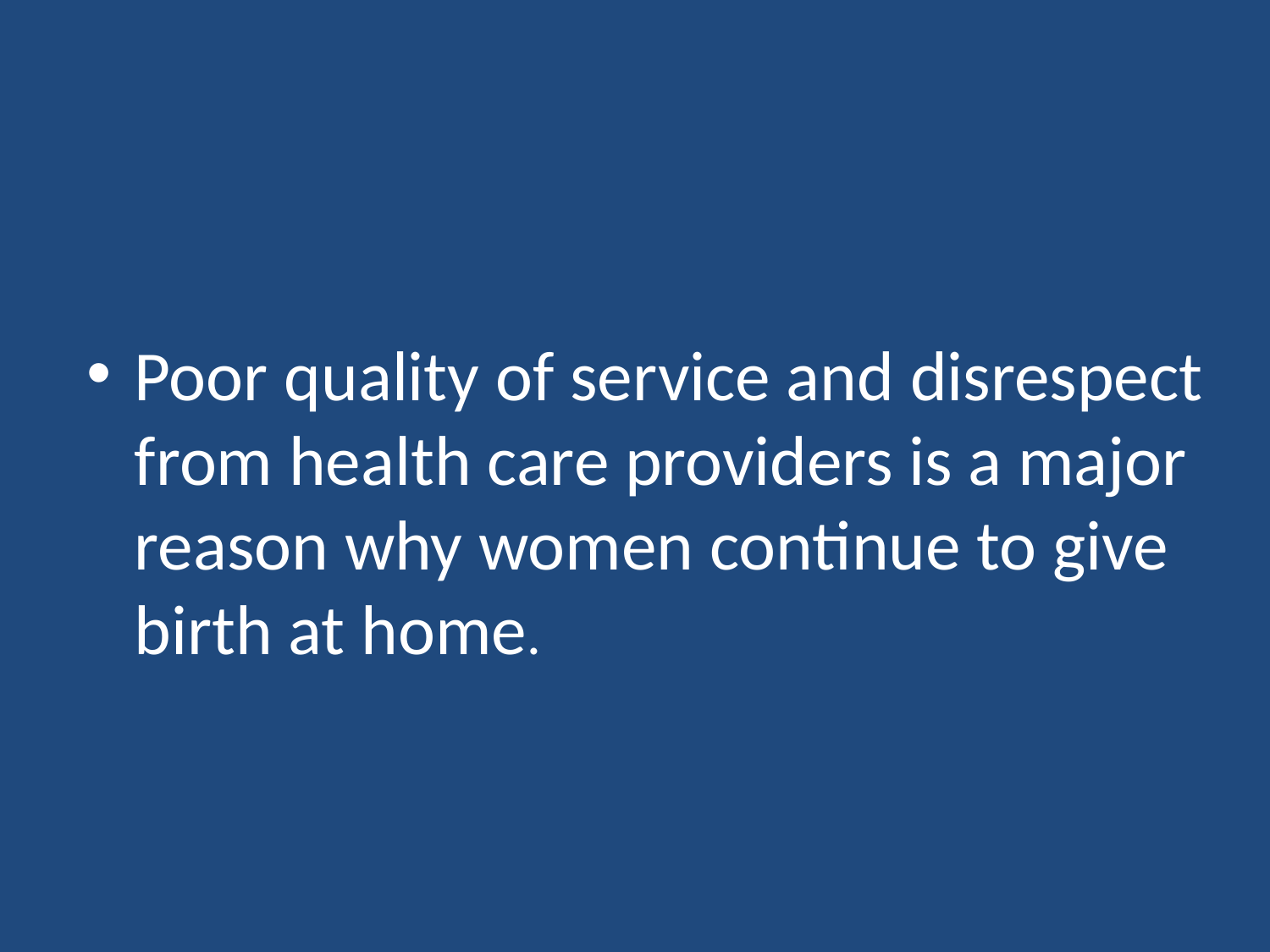

#
Poor quality of service and disrespect from health care providers is a major reason why women continue to give birth at home.

## Slide 10
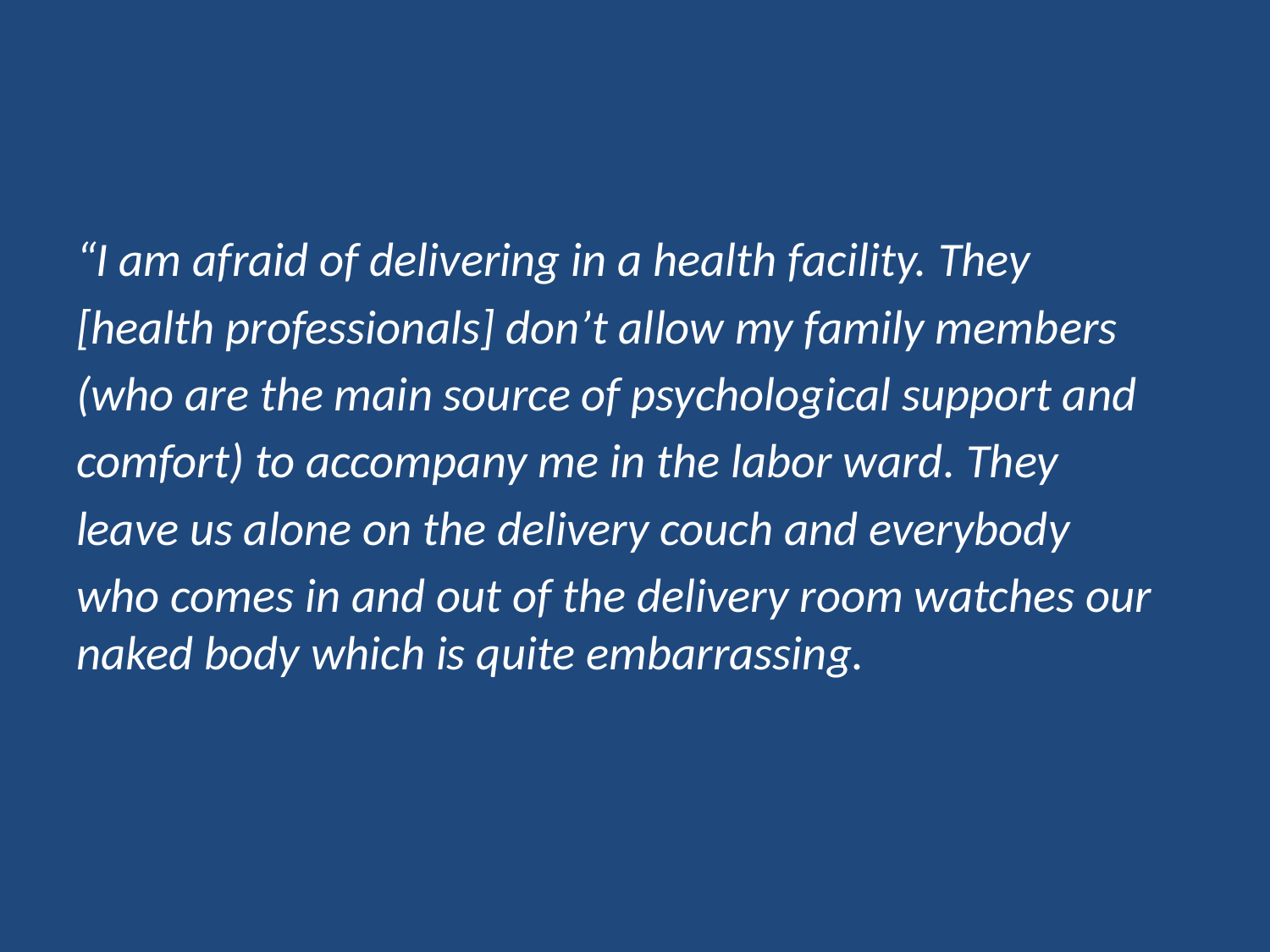

#
“I am afraid of delivering in a health facility. They
[health professionals] don’t allow my family members
(who are the main source of psychological support and
comfort) to accompany me in the labor ward. They
leave us alone on the delivery couch and everybody
who comes in and out of the delivery room watches our naked body which is quite embarrassing.

## Slide 11
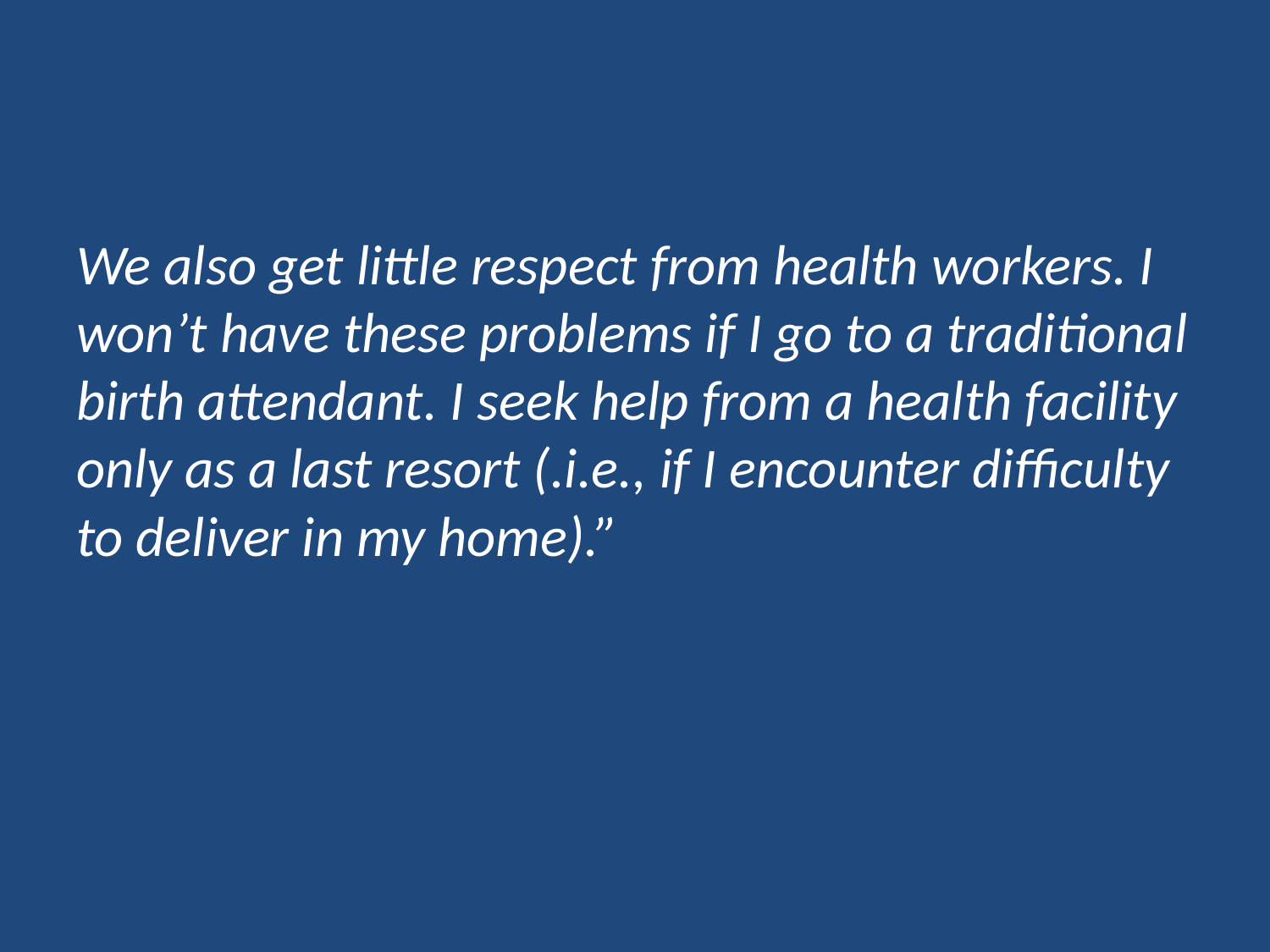

#
We also get little respect from health workers. I won’t have these problems if I go to a traditional birth attendant. I seek help from a health facility only as a last resort (.i.e., if I encounter difficulty to deliver in my home).”

## Slide 12
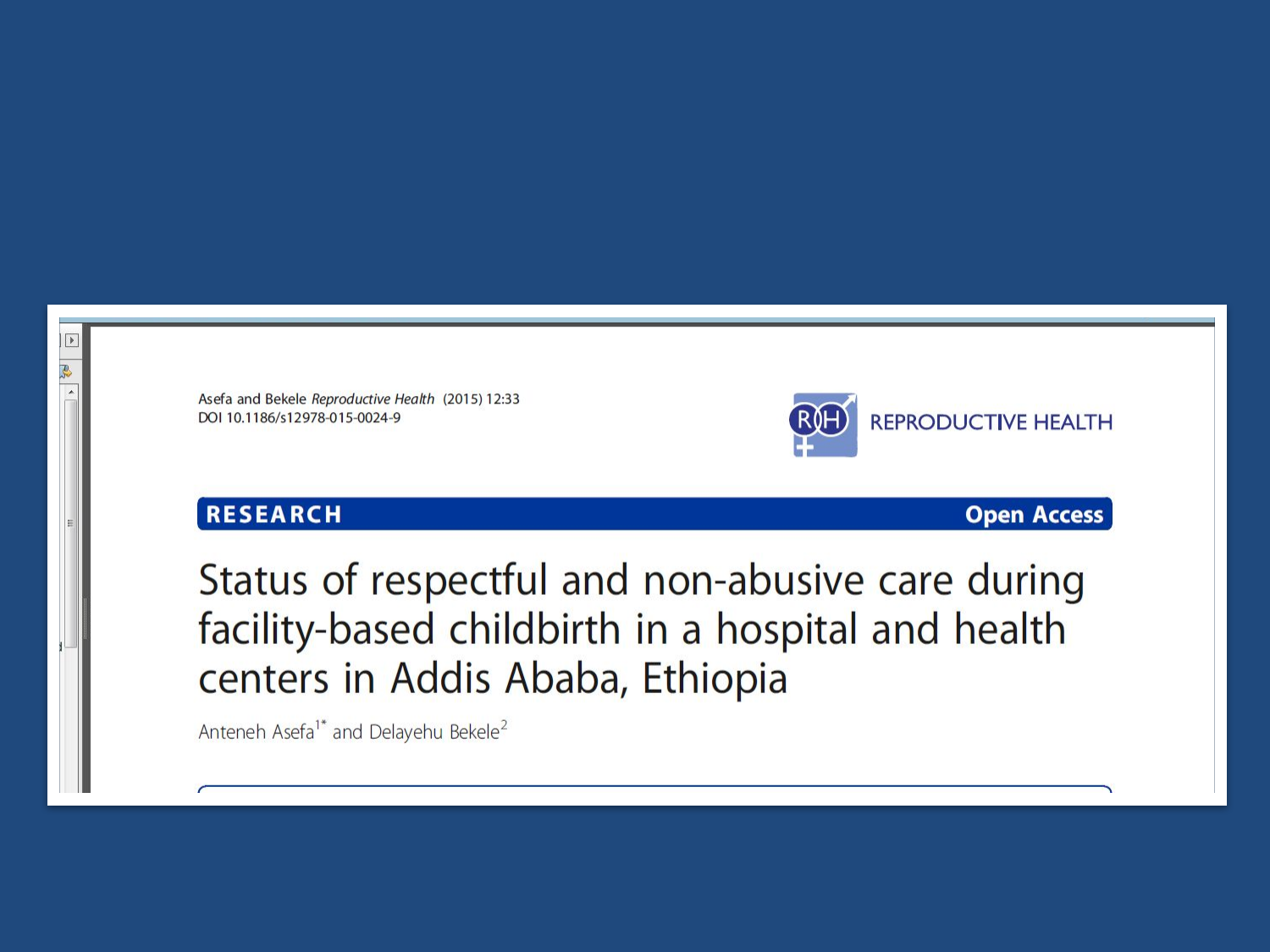

#

## Slide 13
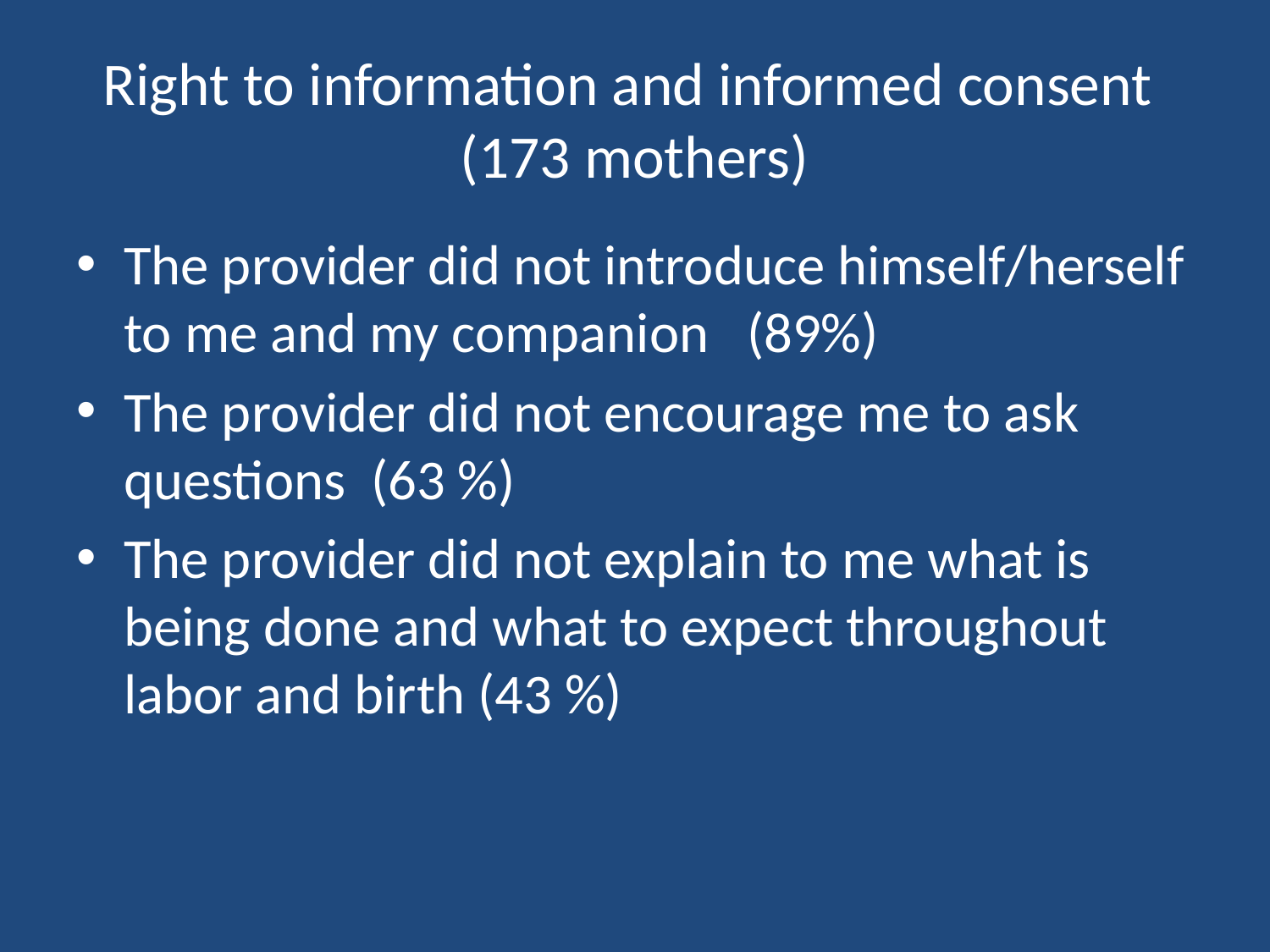

# Right to information and informed consent (173 mothers)
The provider did not introduce himself/herself to me and my companion (89%)
The provider did not encourage me to ask questions (63 %)
The provider did not explain to me what is being done and what to expect throughout labor and birth (43 %)

## Slide 14
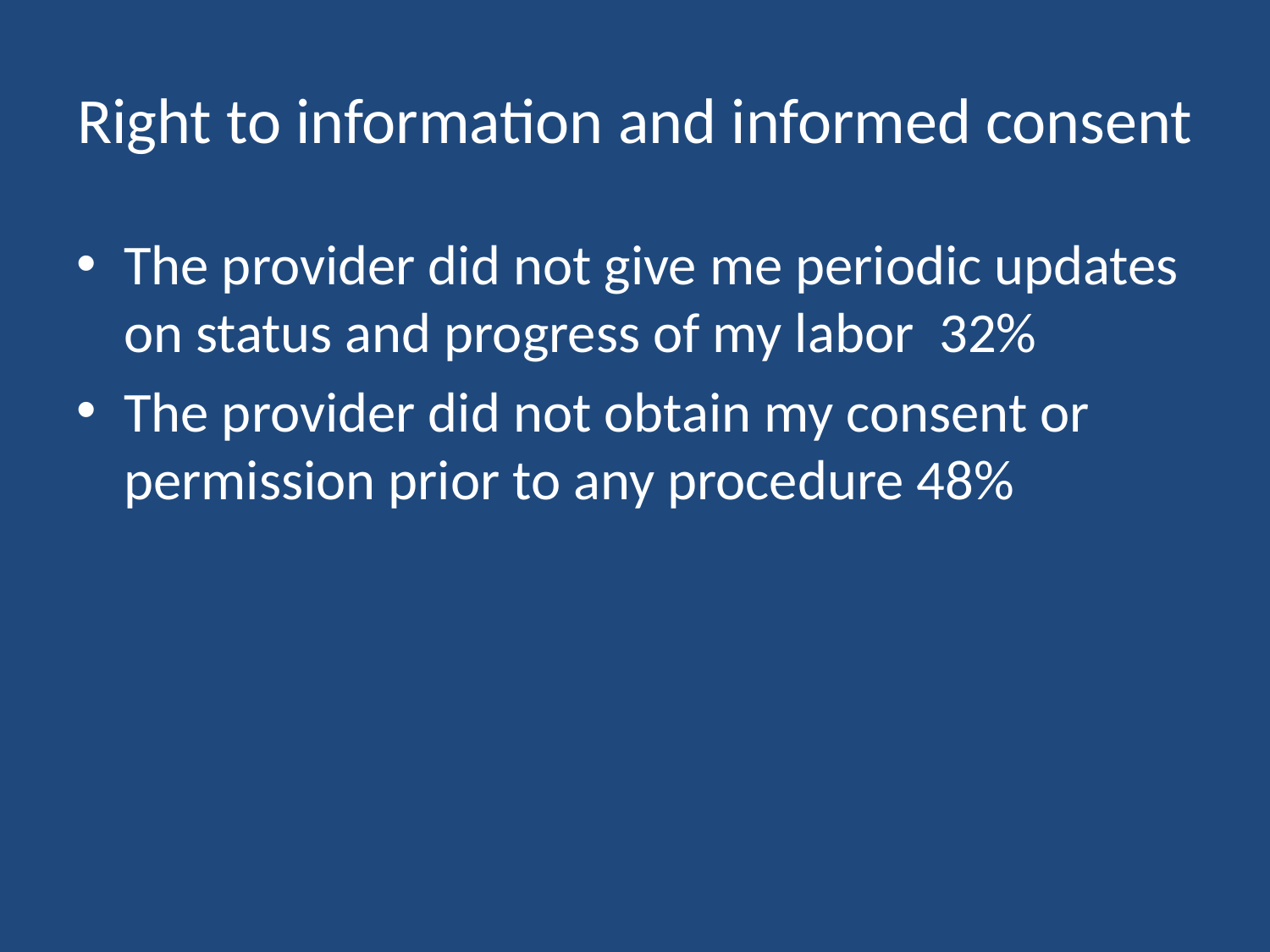

# Right to information and informed consent
The provider did not give me periodic updates on status and progress of my labor 32%
The provider did not obtain my consent or permission prior to any procedure 48%

## Slide 15
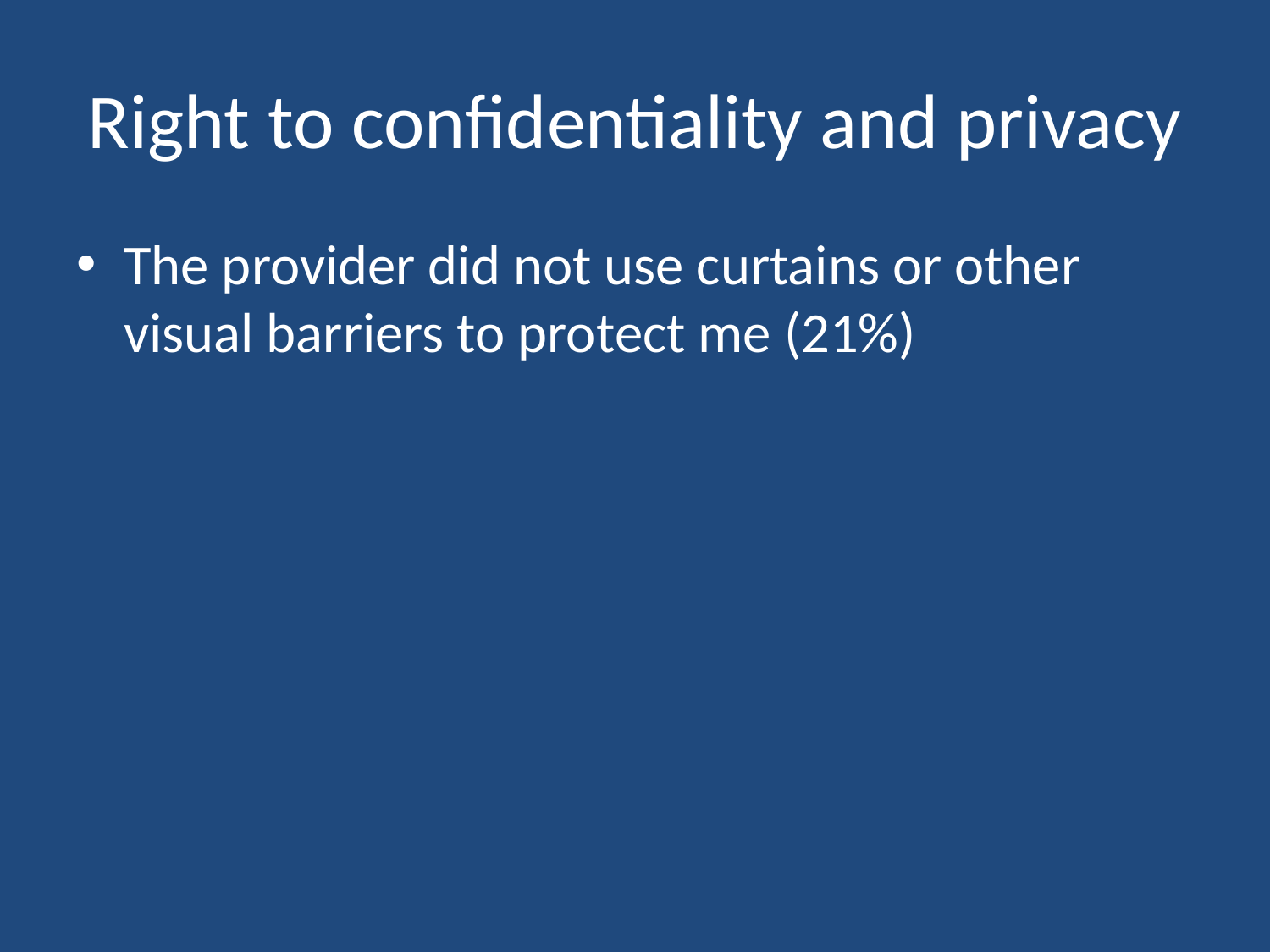

# Right to confidentiality and privacy
The provider did not use curtains or other visual barriers to protect me (21%)

## Slide 16
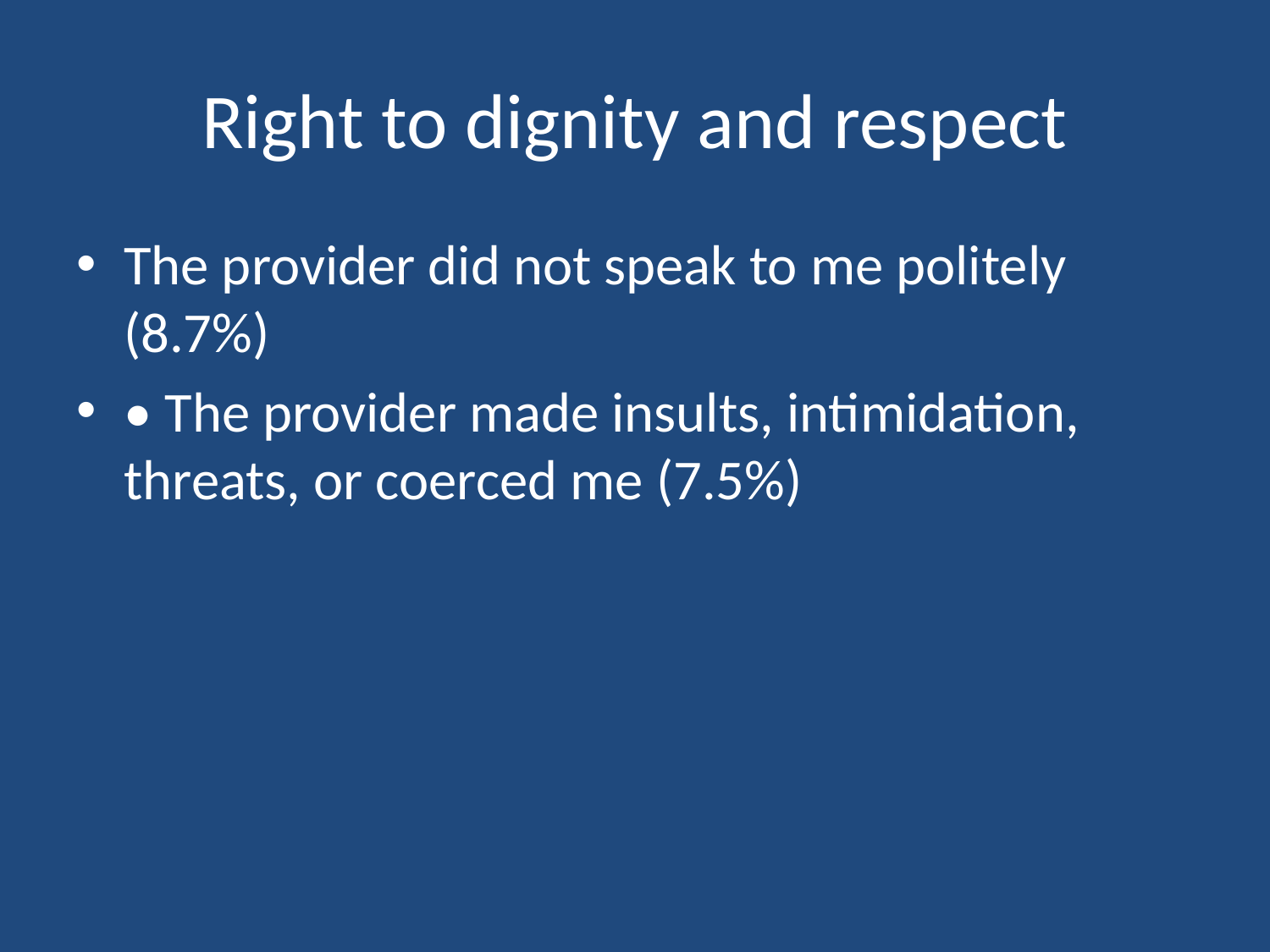

# Right to dignity and respect
The provider did not speak to me politely (8.7%)
• The provider made insults, intimidation, threats, or coerced me (7.5%)

## Slide 17
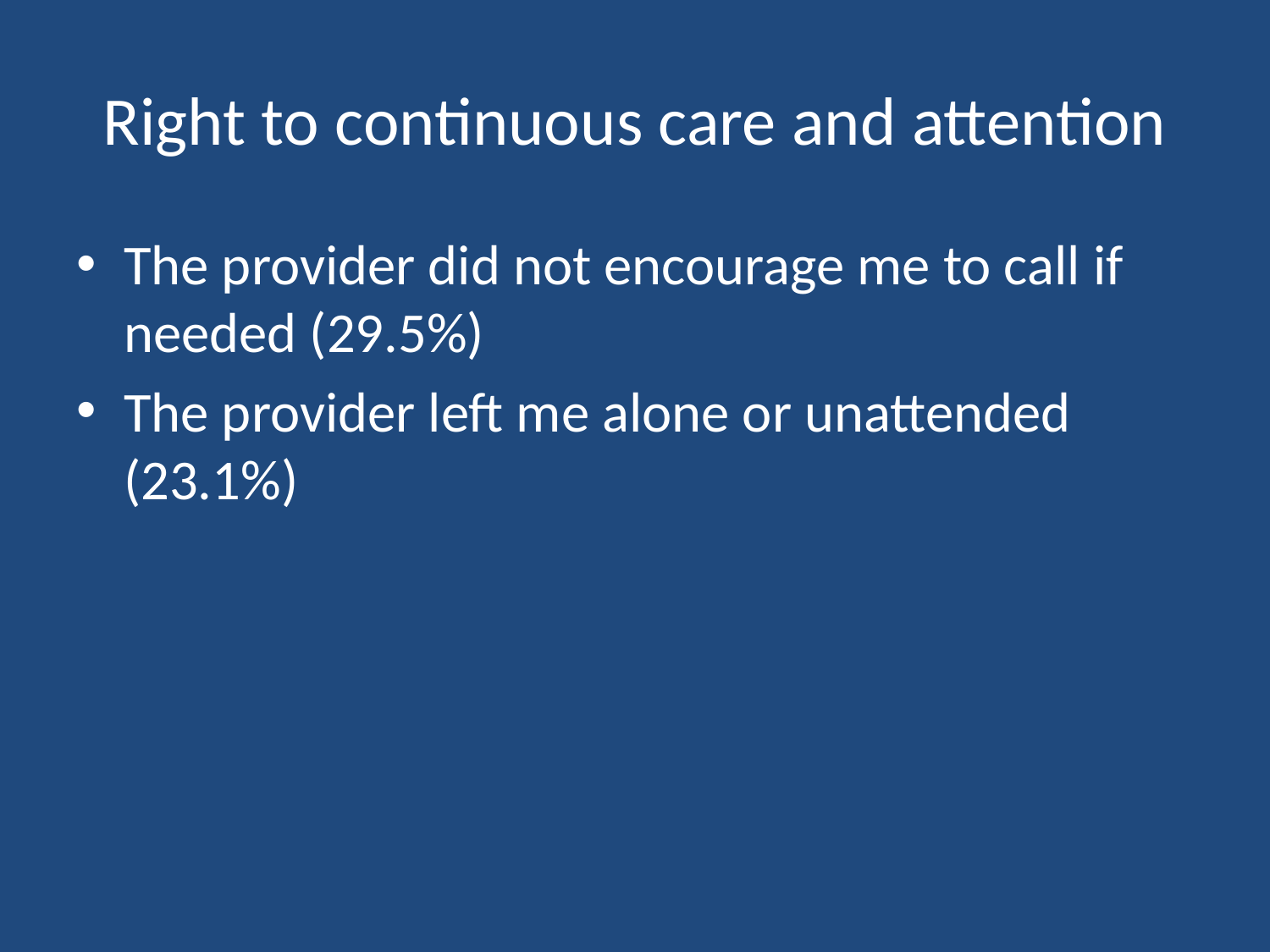

# Right to continuous care and attention
The provider did not encourage me to call if needed (29.5%)
The provider left me alone or unattended (23.1%)

## Slide 18
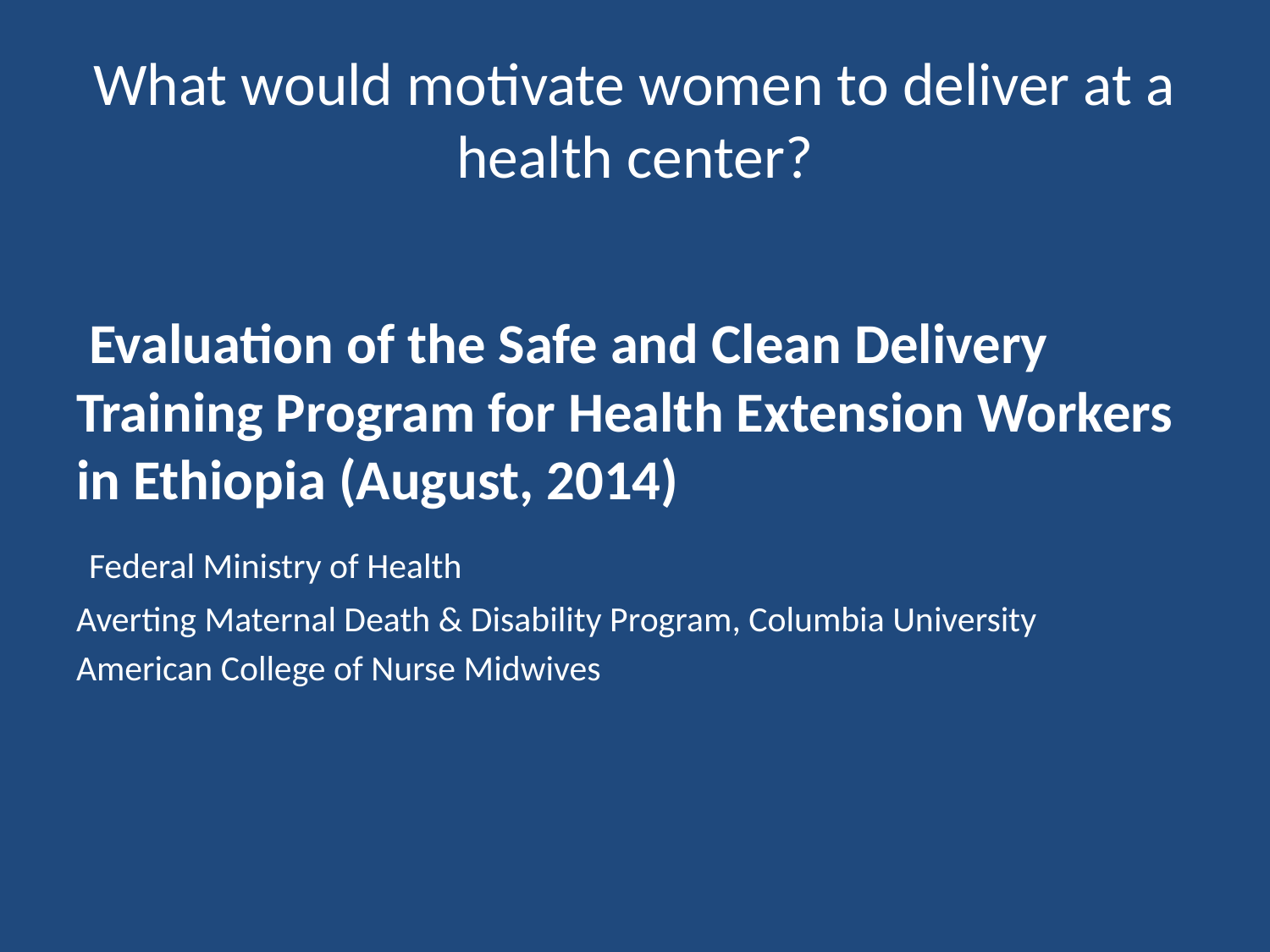

# What would motivate women to deliver at a health center?
 Evaluation of the Safe and Clean Delivery Training Program for Health Extension Workers in Ethiopia (August, 2014)
 Federal Ministry of Health
Averting Maternal Death & Disability Program, Columbia University
American College of Nurse Midwives

## Slide 19
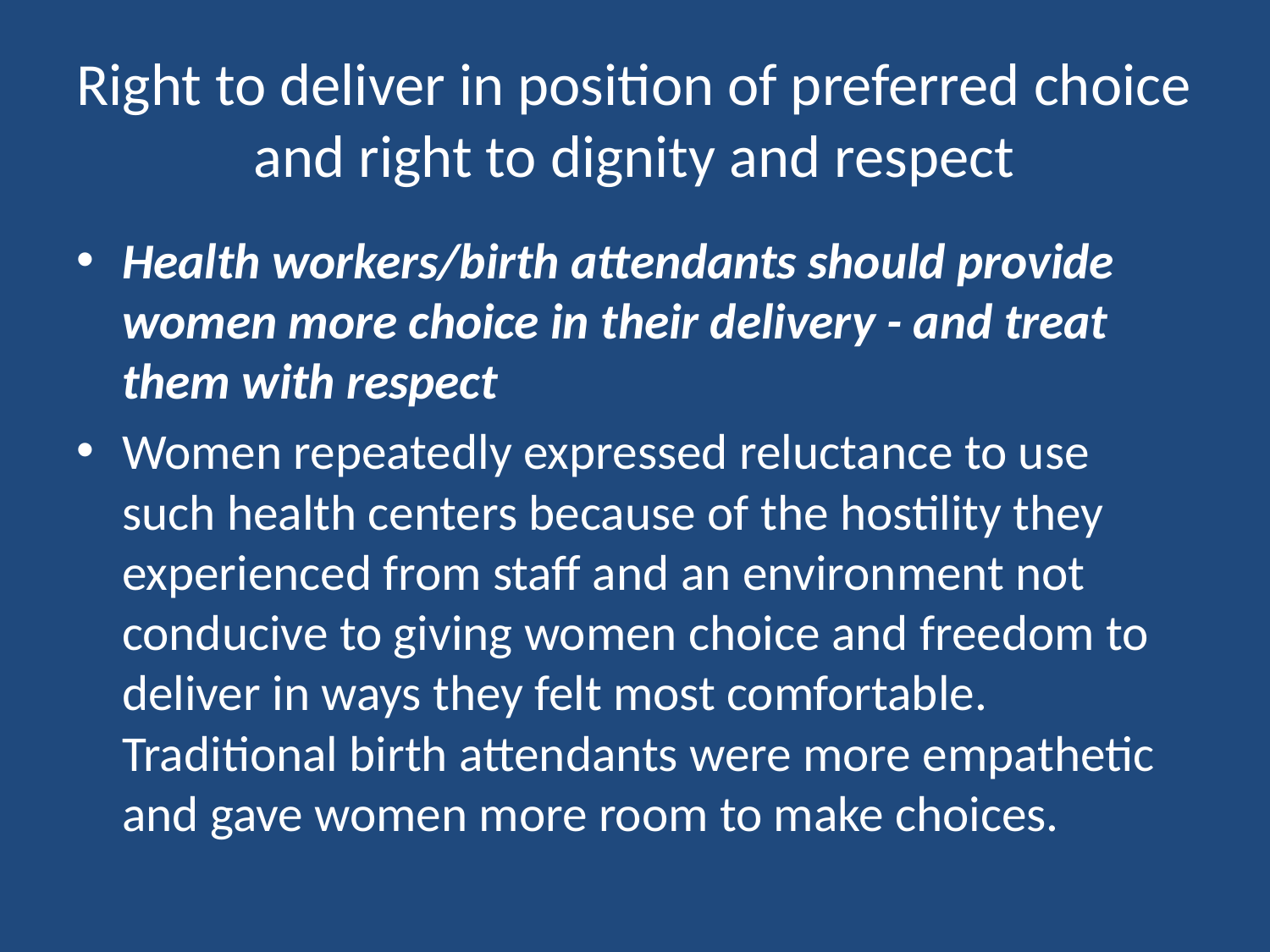

# Right to deliver in position of preferred choice and right to dignity and respect
Health workers/birth attendants should provide women more choice in their delivery - and treat them with respect
Women repeatedly expressed reluctance to use such health centers because of the hostility they experienced from staff and an environment not conducive to giving women choice and freedom to deliver in ways they felt most comfortable. Traditional birth attendants were more empathetic and gave women more room to make choices.

## Slide 20
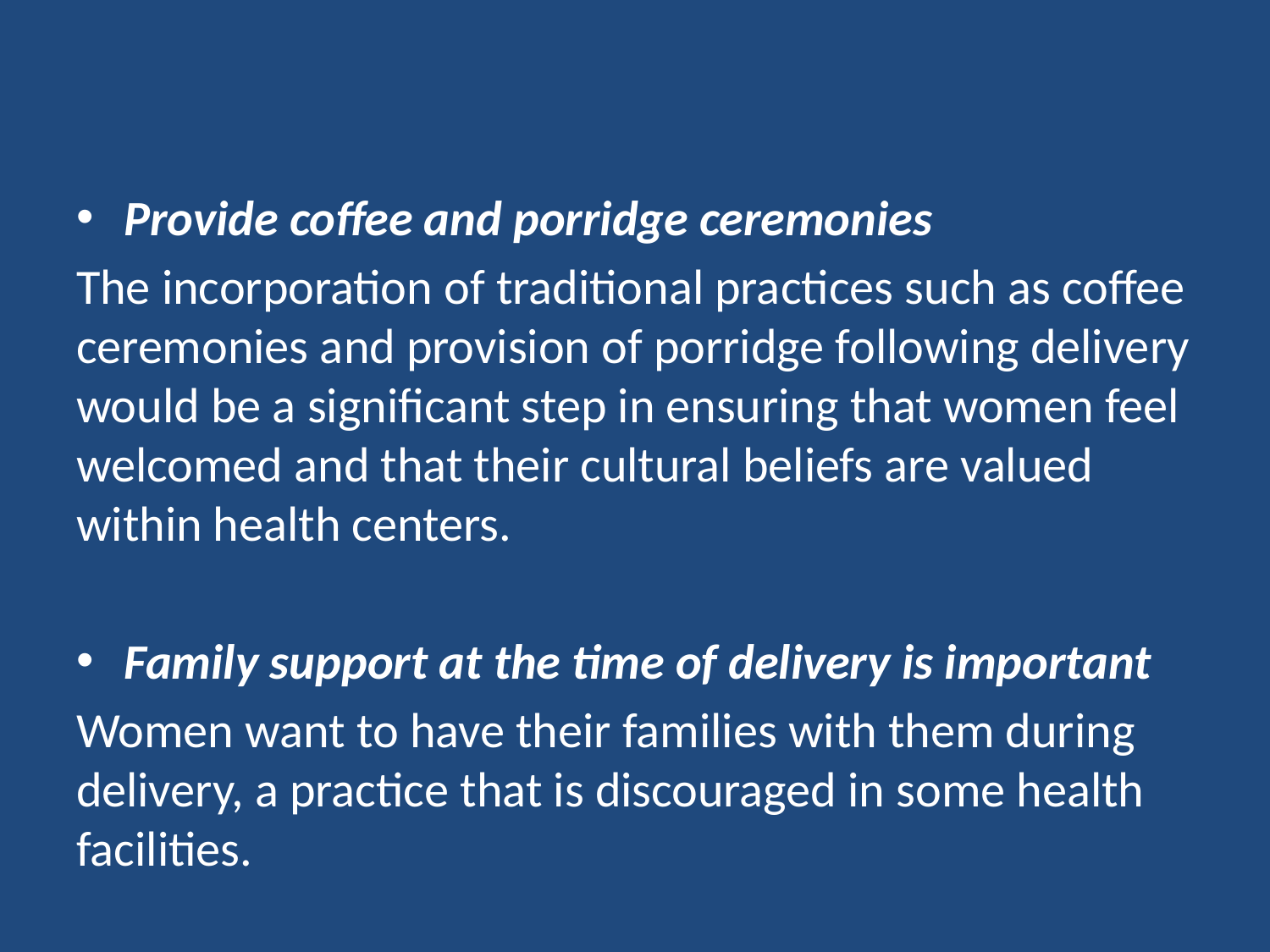

Provide coffee and porridge ceremonies
The incorporation of traditional practices such as coffee ceremonies and provision of porridge following delivery would be a significant step in ensuring that women feel welcomed and that their cultural beliefs are valued within health centers.
Family support at the time of delivery is important
Women want to have their families with them during delivery, a practice that is discouraged in some health facilities.

## Slide 21
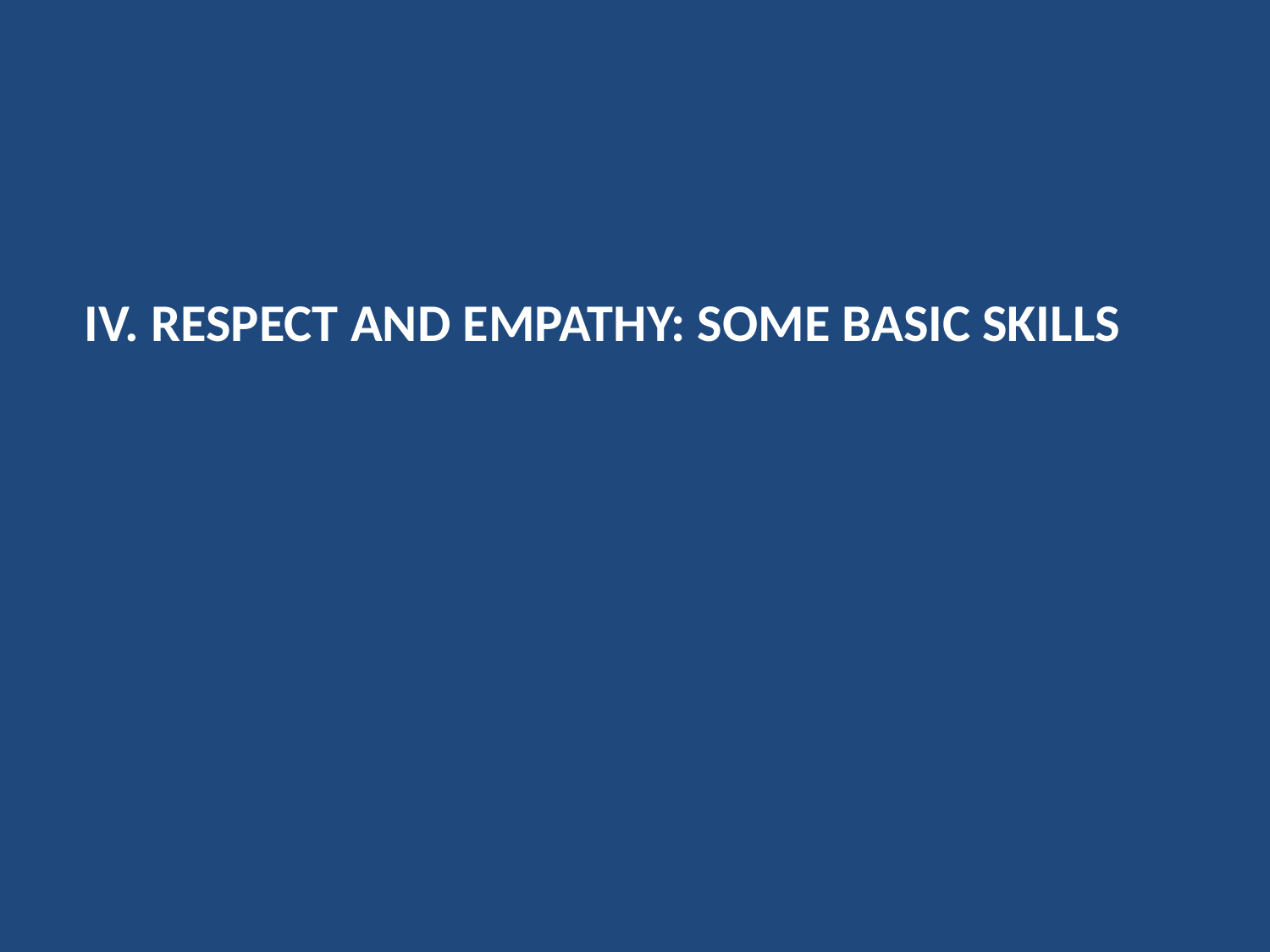

# iV. Respect and empathy: some basic skills

## Slide 22
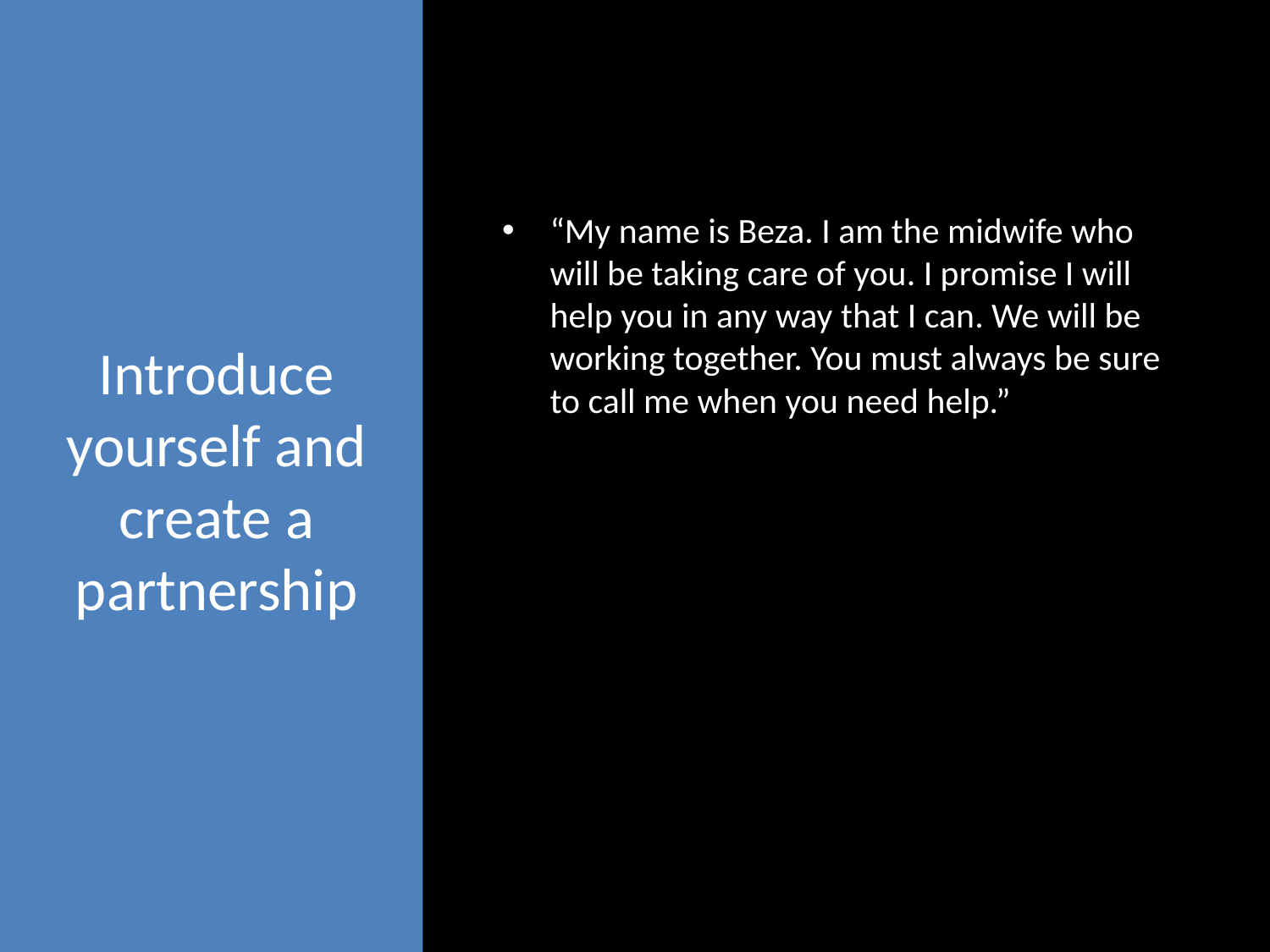

# Introduce yourself and create a partnership
“My name is Beza. I am the midwife who will be taking care of you. I promise I will help you in any way that I can. We will be working together. You must always be sure to call me when you need help.”

## Slide 23
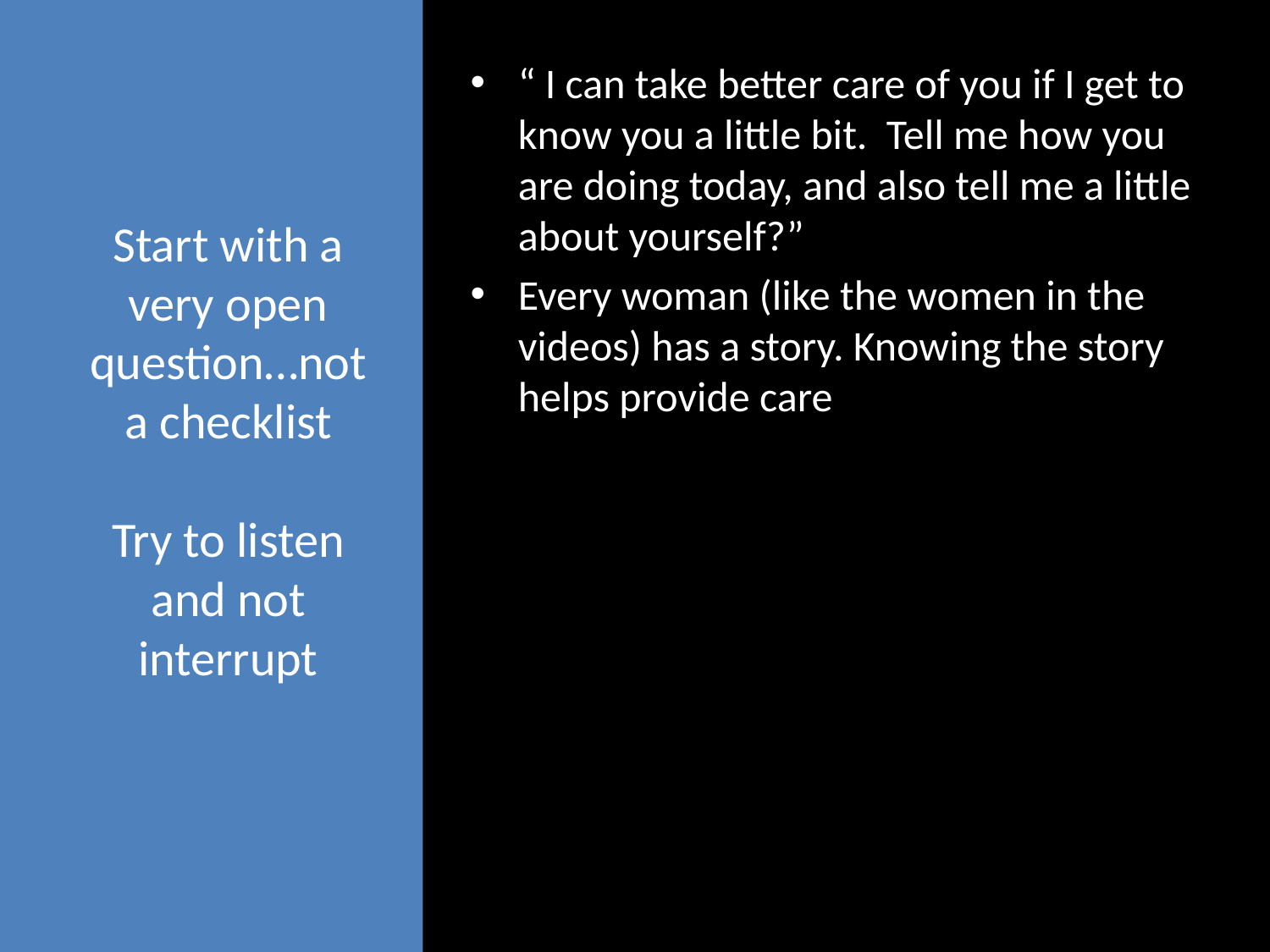

“ I can take better care of you if I get to know you a little bit. Tell me how you are doing today, and also tell me a little about yourself?”
Every woman (like the women in the videos) has a story. Knowing the story helps provide care
# Start with a very open question…not a checklist Try to listen and not interrupt

## Slide 24
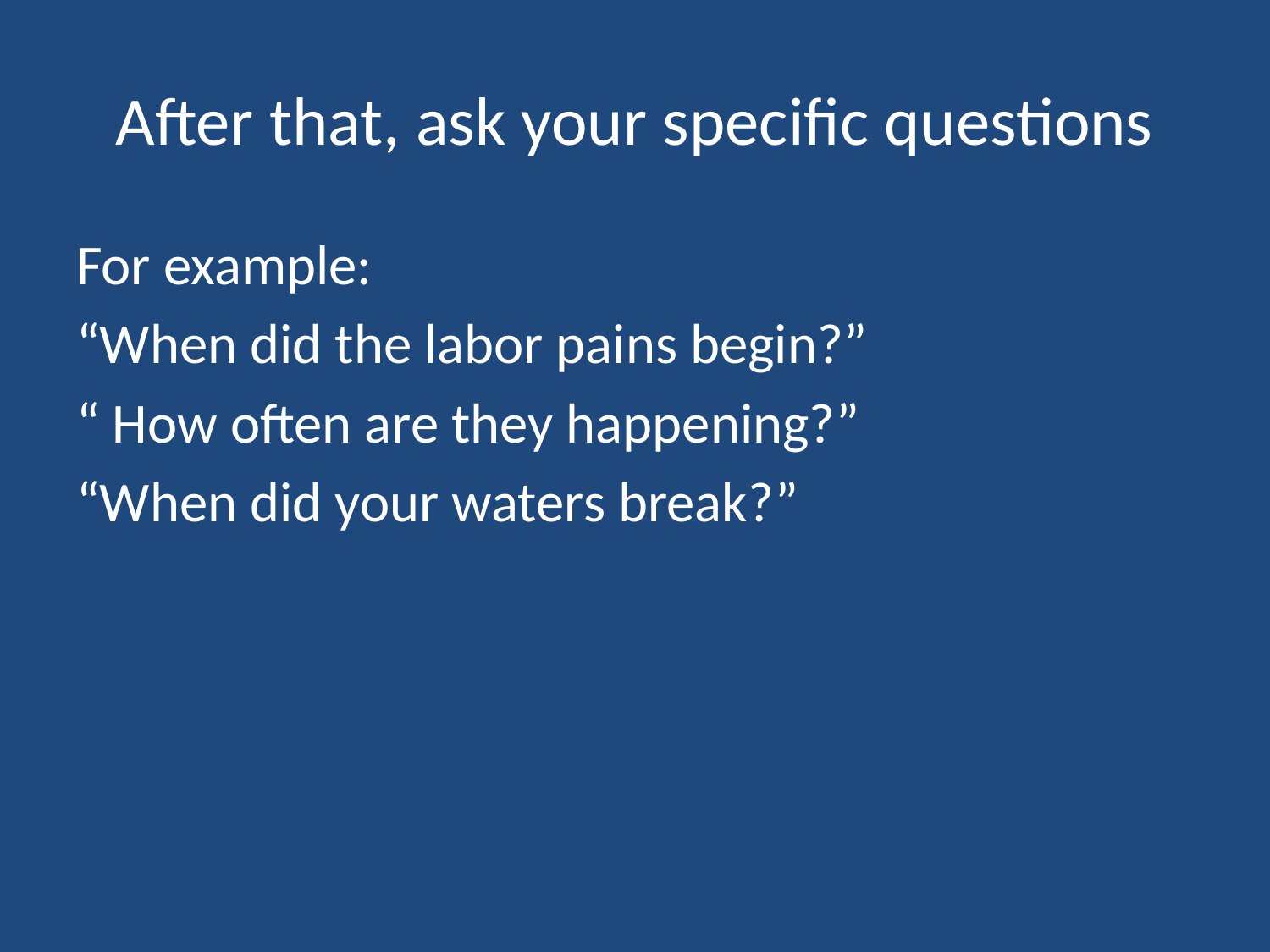

# After that, ask your specific questions
For example:
“When did the labor pains begin?”
“ How often are they happening?”
“When did your waters break?”

## Slide 25
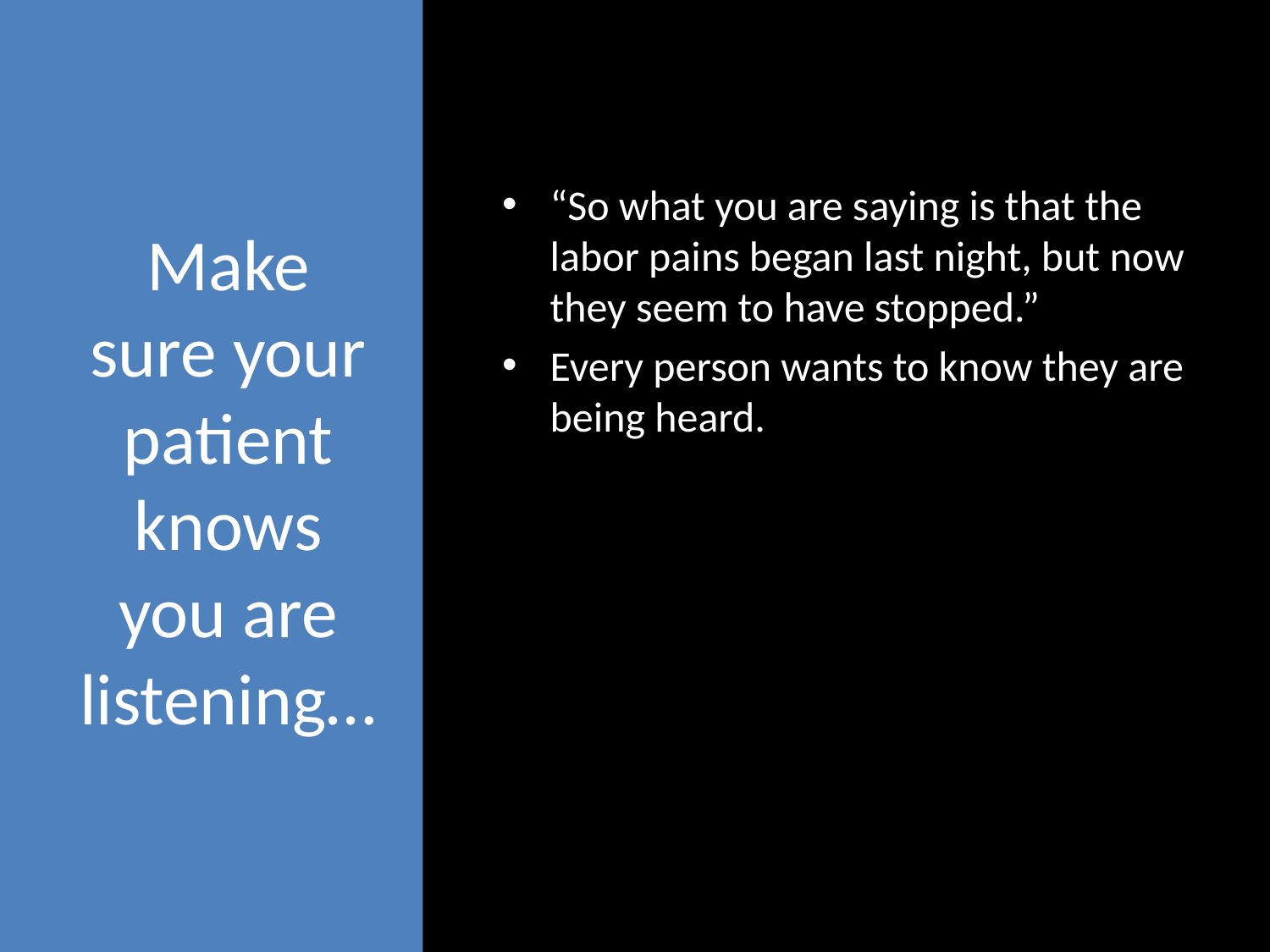

# Make sure your patient knows you are listening…
“So what you are saying is that the labor pains began last night, but now they seem to have stopped.”
Every person wants to know they are being heard.

## Slide 26
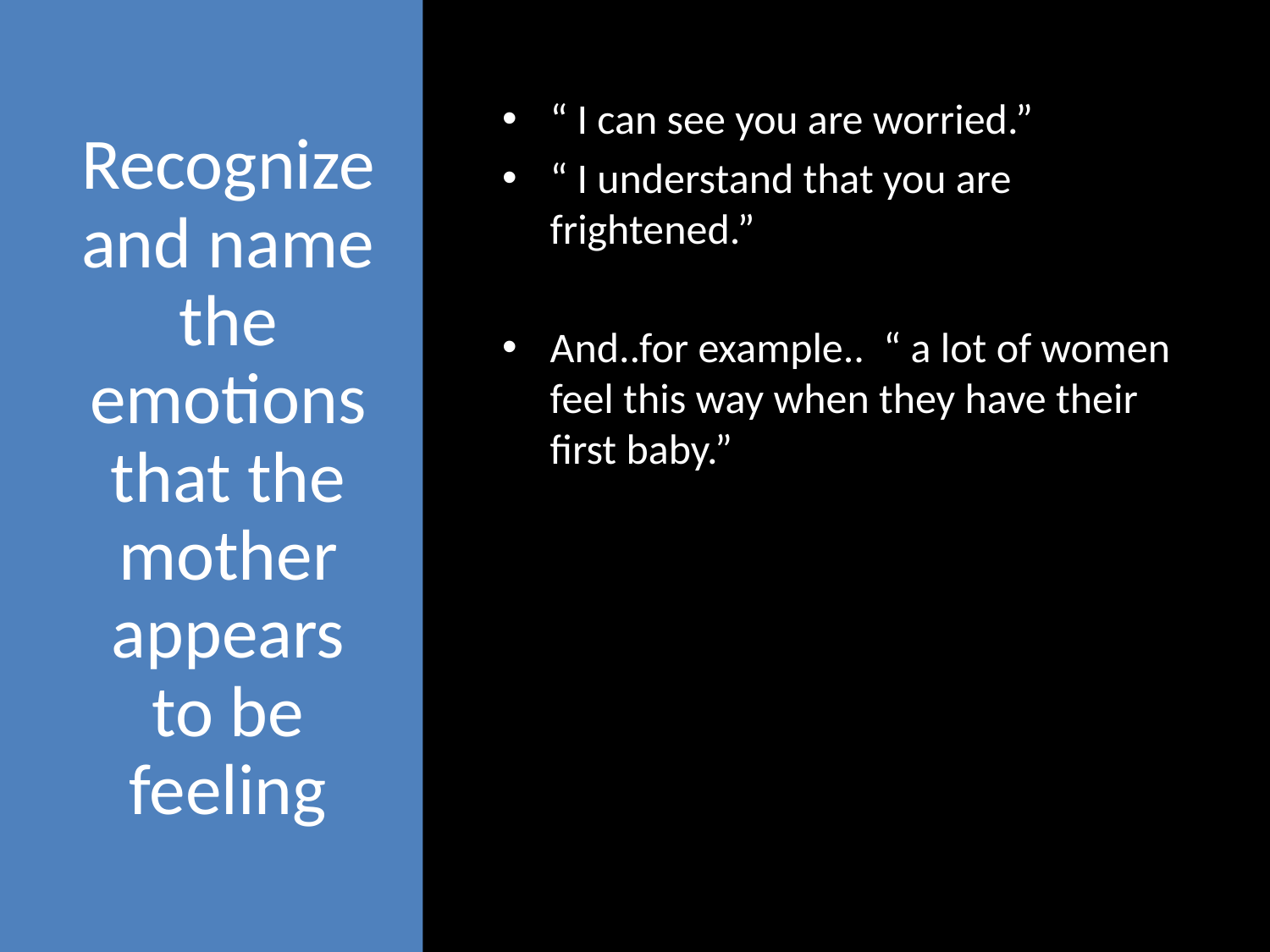

# Recognize and name the emotions that the mother appears to be feeling
“ I can see you are worried.”
“ I understand that you are frightened.”
And..for example.. “ a lot of women feel this way when they have their first baby.”

## Slide 27
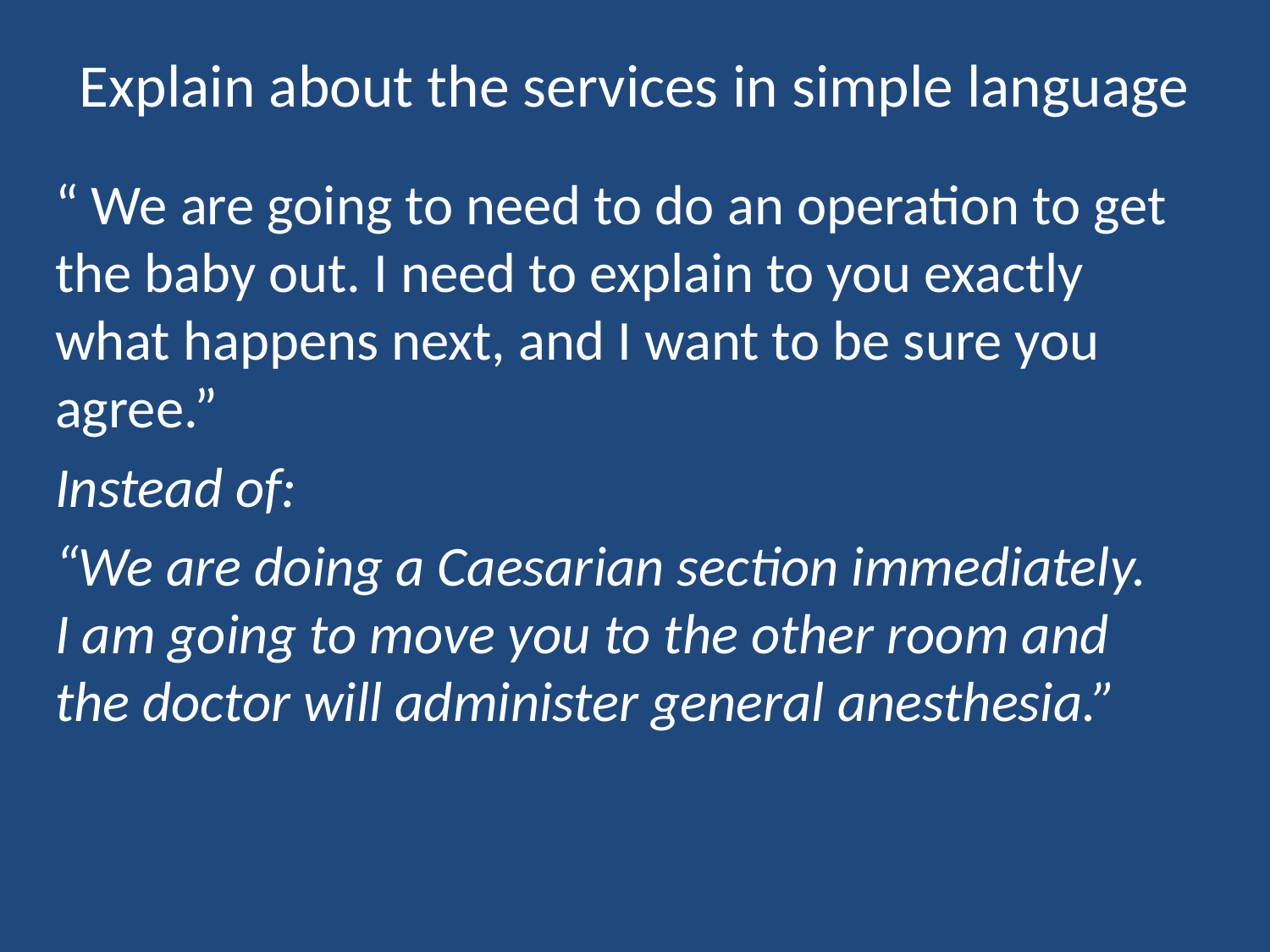

# Explain about the services in simple language
“ We are going to need to do an operation to get the baby out. I need to explain to you exactly what happens next, and I want to be sure you agree.”
Instead of:
“We are doing a Caesarian section immediately. I am going to move you to the other room and the doctor will administer general anesthesia.”

## Slide 28
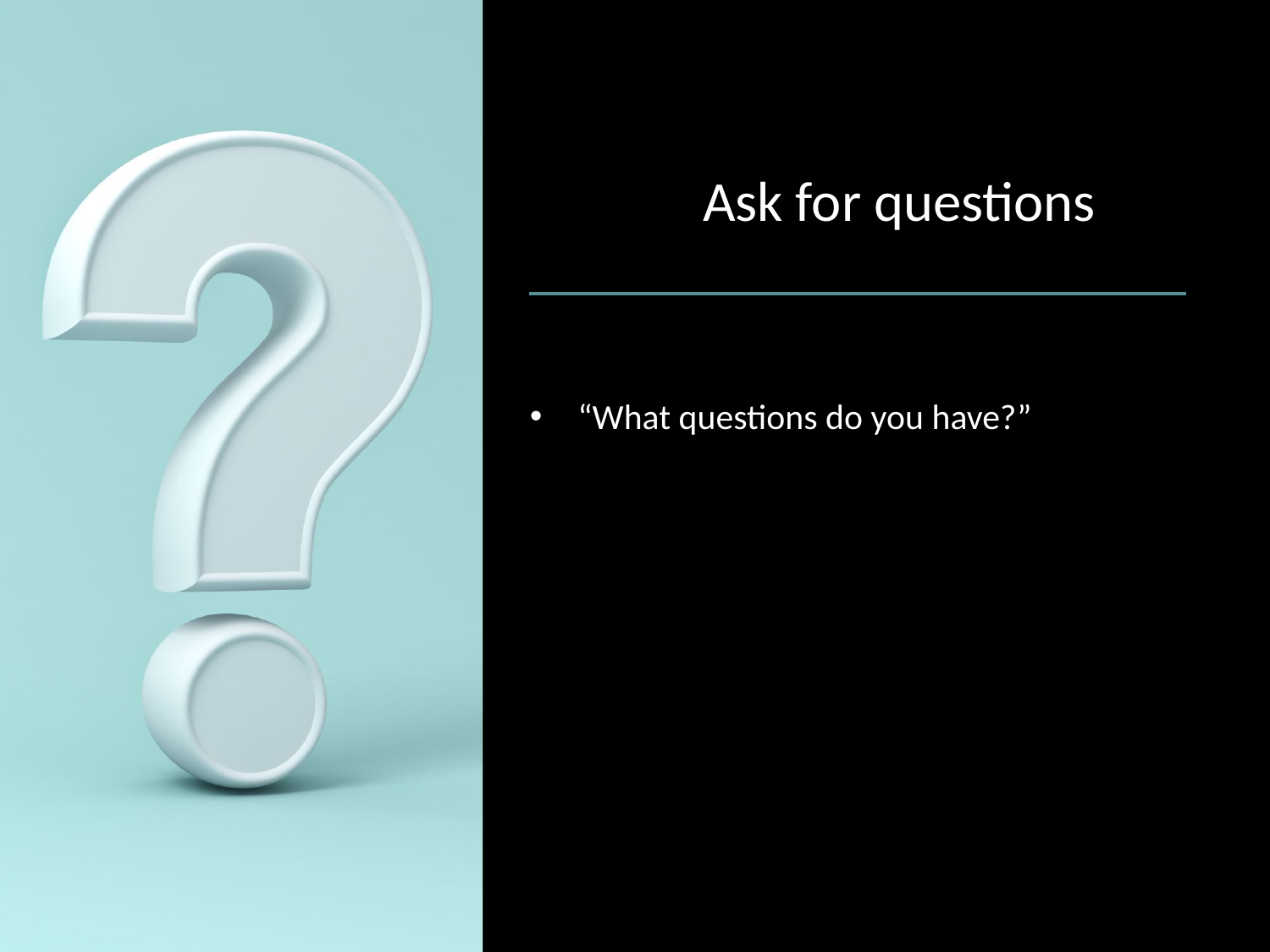

Ask for questions
“What questions do you have?”

## Slide 29
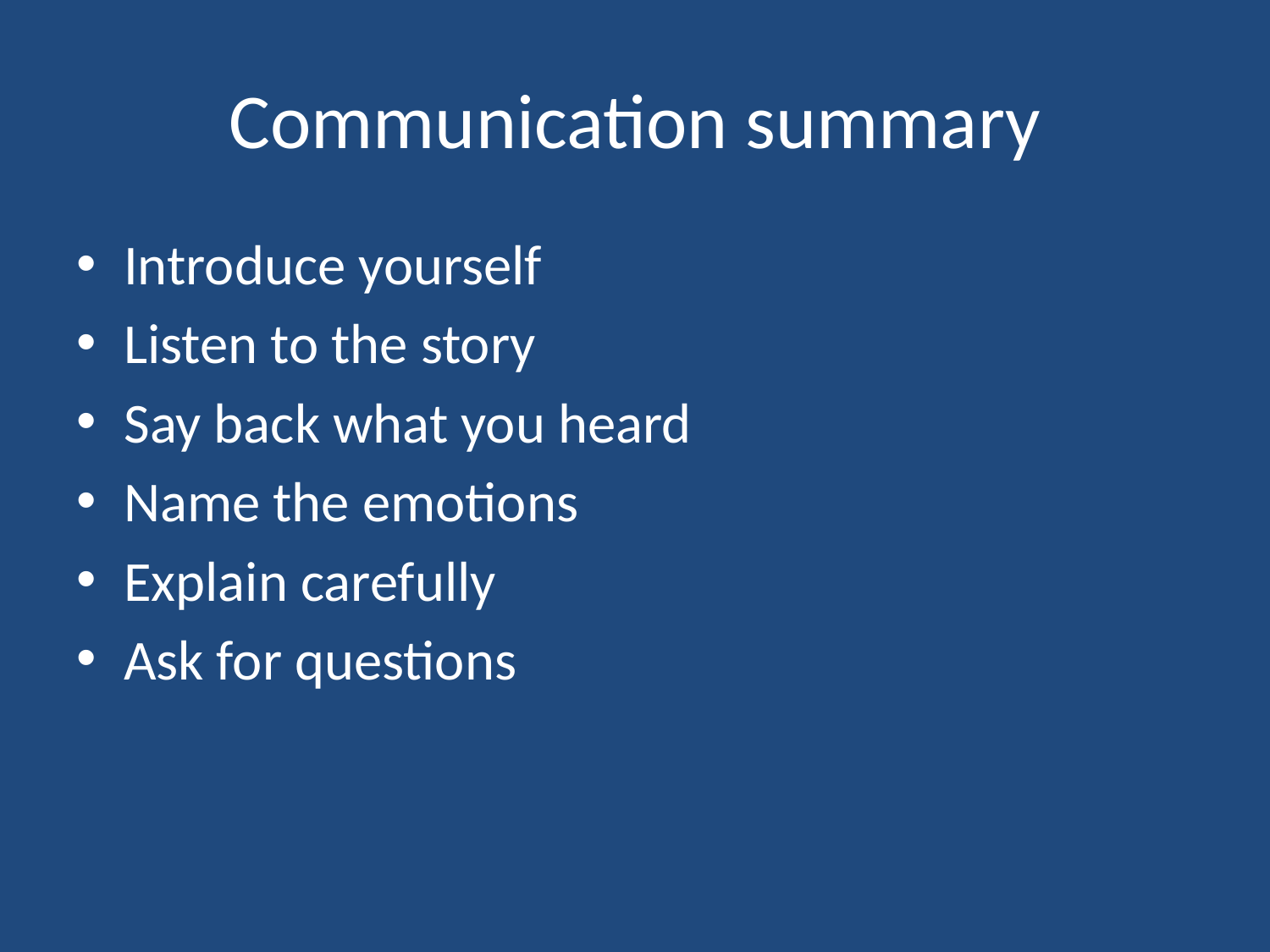

# Communication summary
Introduce yourself
Listen to the story
Say back what you heard
Name the emotions
Explain carefully
Ask for questions

## Slide 30
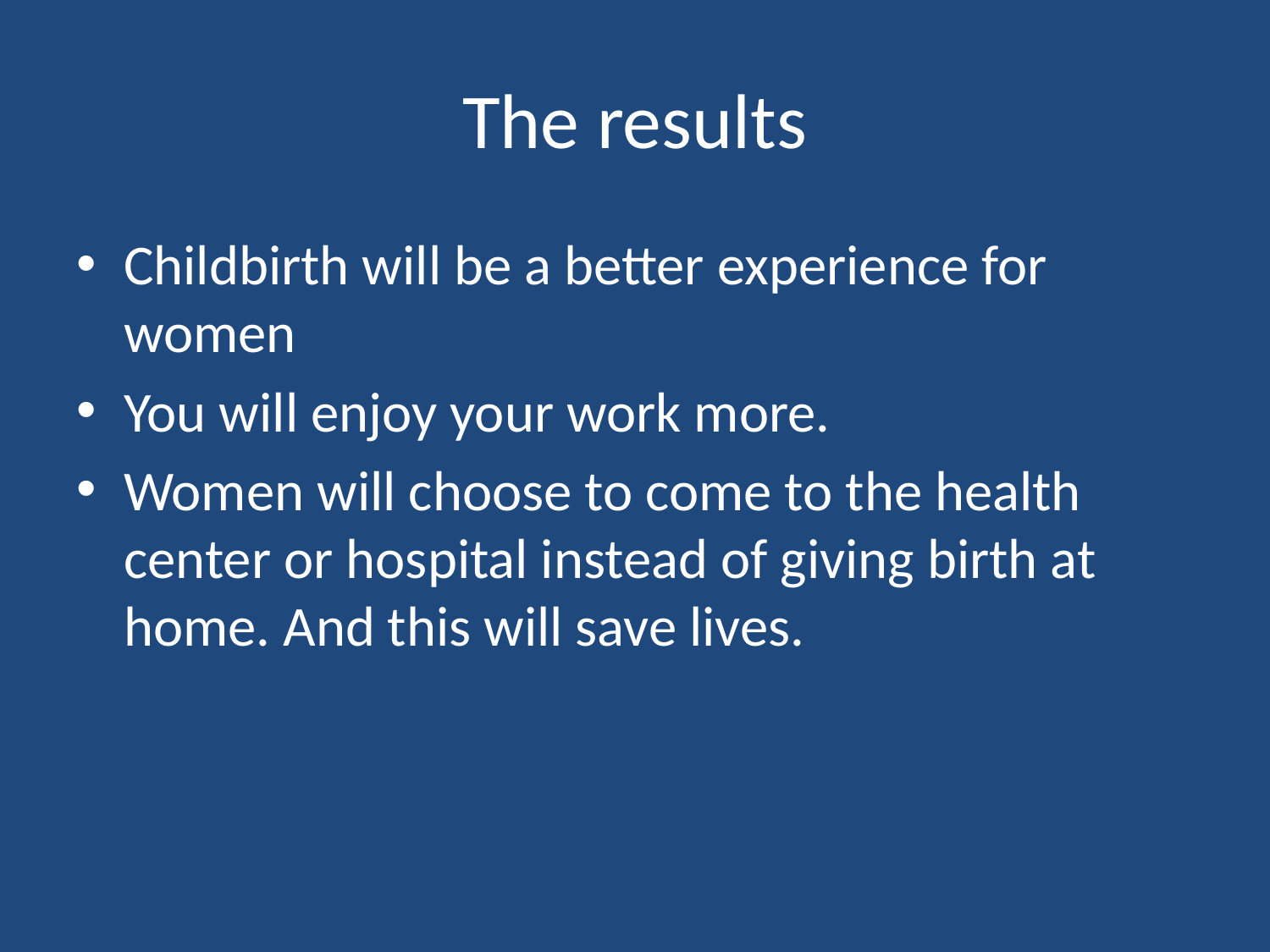

# The results
Childbirth will be a better experience for women
You will enjoy your work more.
Women will choose to come to the health center or hospital instead of giving birth at home. And this will save lives.
