# Supplementary material for: An innovative intervention to improve respectful maternity care in three Districts in Ethiopia
Source: BMC Pregnancy Childbirth. 2021 Aug 6;21:541. doi: 10.1186/s12884-021-03934-y (PMC8343890; doi:10.1186/s12884-021-03934-y)
Supplement: Supplementary file 3 — Additional file 3. Safe Childbirth Checklist adopted by the Ethiopian Ministry of Health. [file 12884_2021_3934_MOESM3_ESM.pdf]

Before Birth | SAFE CHILDBIRTH CHECKLIST

1. On admission

Quick check performed?

☐ Yes

☐ No

- Assess for danger signs

Does mother need referral?

☐ Yes, organized with initial stabilization and treatment according to signs

☐ No

Refer to a higher level if any of the following danger signs are present (if the setup and professional is not present):
- Vaginal bleeding
- Severe abdominal pain
- High fever > 37.5°C
- Difficulty in breathing
- History of heart disease or other major illnesses
- Severe headache or blurred vision
- Convulsions

Partograph started?

☐ Yes

☐ No, will start when ≥ 4 cm

Start plotting when cervix ≥ 4 cm—on alert line.
- Every 30 min: plot maternal pulse, contractions, fetal heart rate
- Every 2 hours: plot temperature
- Every 4 hours: plot blood pressure
- Every 4 hours: vaginal examination

Does mother need to start:

Antibiotics?

☐ Yes, IV started

☐ No

Give if:
- Temperature > 37.5°C
- Foul-smelling vaginal discharge
- Rupture of membranes >12 hours,
- OR labor >24 hours

Magnesium sulfate?

☐ Yes, given

☐ No

Give if:
- Convulsions
- Diastolic blood pressure ≥110 mm Hg and 3+ proteinuria (give antihypertensive) OR
- Diastolic blood pressure ≥90 mm Hg, 2+ proteinuria, and any: severe headache, visual disturbance, or epigastric pain or oliguria, pulmonary edema
- Diastolic blood pressure ≥90 mm Hg, 2+ proteinuria in labor

Antiretroviral medicine?

☐ Yes, given

☐ No, confirmed HIV negative

☐ If status unknown, HIV test done

Give ART if mother is HIV+
If on ART, continue

Are soap, water, alcohol hand rub, gloves available?

☐ Yes, I will wash hands and wear gloves for each vaginal exam

☐ No, arrange supplies

☐ **Confirm if birth companion encouraged to be present throughout labor and at birth**

☐ **Confirm that mother/companion will call for help during labor if mother has a danger sign**

Call for help if:
- Bleeding
- Severe abdominal pain
- Severe headache or blurring of vision
- Convulsions
- Urge to push
- Difficulty emptying bladder

☐ **Confirm mothers privacy is maintained during labour and delivery**

2. Just before second stage/birth of baby (or before cesarean)

Does mother need to start antibiotics?

☐ Yes, IV started

☐ No

Give if:
- Temperature >37.5°C
- Foul-smelling vaginal discharge
- Rupture of membranes > 12 hours
- Labor > 24 hours
- Before cesarean section

Does mother need to start magnesium sulfate?

☐ Yes, given

☐ No

Give if:
- Convulsions
- Diastolic blood pressure ≥ 110 mm Hg and 3+ proteinuria, (give antihypertensive) OR
- Diastolic blood pressure ≥ 90 mm Hg, 2+ proteinuria, and any: severe headache, visual disturbance, or epigastric pain or oliguria, pulmonary edema
- Diastolic blood pressure ≥ 90 mm Hg, 2+ proteinuria in labor

Are essential supplies at bedside for mother?

☐ Gloves

☐ Soap/Savlon and clean water

☐ Oxytocin 10 IU in syringe

Prepare to care for mother and baby during birth:
- Check for 2nd baby
- Give oxytocin within 1 minute
- Delayed cord clamping in 1–3min
- Deliver placenta by controlled cord traction
- Confirm uterus is contracted

Are essential supplies at bedside for baby?

☐ Two clean, dry, warm towels

☐ Sterile scissors to cut cord

☐ Suction device

☐ Bag-and-mask (size 0 & 1)

☐ Sterile cord tie/clamp

Prepare to care for baby immediately after birth:
- Deliver and dry baby on maternal abdomen, wrap, keep warm, and wipe eyes
- Clamp/tie cord two fingers from abdomen and another two fingers from the first
- Check breathing—If not breathing: stimulate and clear airway
- If still not breathing or if the baby is blue: cut cord, ventilate with bag-and-mask
- Shout for help

Anti-retroviral medicine?

☐ Yes, given

☐ No, confirmed HIV negative

☐ If status unknown, HIV test done

Give ART if mother is HIV+
If on ART, continue

☐ **Confirm assistant identified and informed to be ready to help at birth if needed**

Name of Provider: ……………………..................Date: …………………..Signature: …………..............

Name of Provider: ……………………..................Date: …………………..Signature: …………............. **June 2016 V**

After Birth | SAFE CHILDBIRTH CHECKLIST

3. Soon after birth (within 1 hour)

Does baby need to start antibiotics?

- ☐ Yes, given
- ☐ No

Give if antibiotics were given to mother or if baby has any of following:
- Poor sucking/not sucking
- Chest in-drawing, grunting
- Convulsions
- Poor movement on stimulation
- Too cold (temperature < 35.5°C and not rising after warming) or too hot (temperature >37.5°)

Does baby need referral?

- ☐ Yes, referred
- ☐ No

Refer to NICU if:
- Any of above criteria or
- Jaundice or pallor

Does baby need special care and monitoring?

- ☐ Yes, special care arranged
- ☐ No

Arrange special care if:
- More than 1 month (4 weeks) early,
- Birth weight < 2,500 grams,
- Needs antibiotics,
- Required resuscitation/HBB

Does baby need to start an anti-retroviral medicine?

- ☐ Yes, given
- ☐ No

If mother is HIV+, give baby Neverapine syrup (prophylaxis to be started within 12 hrs of birth)

Newborn

- ☐ Placed baby in skin-to-skin contact and started breastfeeding within 1 hr (if mother and baby are well)
- ☐ Vitamin K given 1 mg IM on anterior mid-thigh
- ☐ TTC eye ointment given in both eyes
- ☐ Weighed and recorded
- ☐ Give BCG and OPV before discharge

If < 2500 grams, ensure full assessment

☐ **Confirm that mother/companion will call for help if danger signs are present**

DANGER SIGNS

Mother has:
- Bleeding
- Severe abdominal pain
- Severe headache
- Visual disturbance
- Breathing difficulty
- Fever/chills
- Difficulty emptying bladder

Baby has:
- -Fast or difficult breathing
- - Fever
- -Unusually cold
- -Stops feeding well
- -Less activity than normal
- -Yellow discoloration of skin/eyes

4. Before discharge

Is mother's bleeding controlled?

- ☐ Yes
- ☐ No

Does mother need to start antibiotics?

- ☐ Yes, given
- ☐ No

Give if temperature > 37.5°C and any:
- Chills,
- Foul-smelling vaginal discharge.
- Labor > 24hrs at time of delivery

Does baby need to start antibiotics?

- ☐ Yes, given
- ☐ No

Give if:
- Chest in-drawing, grunting,
- Convulsions,
- Poor movement on stimulation,
- Too cold (temperature < 35.5°C and not rising after warming) or too hot (temperature > 37.5°C),
- Poor sucking/not sucking breasts
- Umbilical redness extending to skin or draining pus

If mother HIV+, mother is on ART and baby has Nevirapine syrup for 6 weeks

- ☐ Family planning options discussed and offered to mother
- ☐ Confirm that mother/companion will call for help after discharge if:

DANGER SIGNS

Mother has:
- Bleeding
- Severe abdominal pain
- Severe headache
- Visual disturbance
- Breathing difficulty
- Fever/chills
- Difficulty emptying bladder

Baby has:
- Fast or difficulty breathing
- Fever
- Unusually cold
- Stops feeding well
- Less activity than normal
- Yellow discoloration of skin/eyes

- Follow-up arranged for mother and baby
- Birth notifications from HC to HPs for PNC through call/written.

- Refer mother to three postnatal visits (6–24 hours, 3
- Days, 7 days) and an immunization visit at 6 weeks

Name of provider: ……………………..................Date: …………………..Signature: ………….............. Name of Provider: ……………………..................Date: …………………..Signature: …………..............
